# Humidity‐Guided In Situ Polymerization for Controlled Formation of Smooth, Oriented Covalent Organic Framework Films

**DOI:** 10.1002/smll.74867

**Published:** 2026-07-31

**Authors:** Mitsuharu Suzuki, Sora Yamazaki, Yoshiki Shirota, Satoshi Haze, Junya Yasaka, Ryunosuke Sakamoto, Naoya Aizawa, Osamu Ikeda, Tomoyuki Koganezawa, Rosantha Kumara, Ken‐ichi Nakayama

**Affiliations:** ^1^ Division of Applied Chemistry Graduate School of Engineering The University of Osaka Suita Osaka Japan; ^2^ Department of Applied Chemistry Division of Applied Science School of Engineering The University of Osaka Suita Osaka Japan; ^3^ Institute for Chemical Reaction Design and Discovery (WPI‐ICReDD) Hokkaido University Sapporo Hokkaido Japan; ^4^ Japan Synchrotron Radiation Research Institute (JASRI) Sayo‐gun Hyogo Japan

**Keywords:** covalent organic frameworks, morphology, polymerization, sensors, thin films

## Abstract

Efficient, reliable preparation of high‐quality films of covalent organic frameworks (COFs) is essential for fully exploring the potential of this class of porous crystalline compounds in, for example, sensing and catalysis. To this end, in situ polymerization on substrates offers a promising route; however, it remains a challenge to achieve highly crystalline, smooth, and oriented COF films through this method. Here, we present a humidity‐controlled, drop‐casting‐based approach for the formation of imine‐COF films, using the monomer pair 1,3,6,8‐tetrakis(4‐aminophenyl)pyrene and terephthalaldehyde as a representative example, along with additional pairs to highlight the method's versatility. This method affords homogeneous, face‐on‐oriented films with excellent reproducibility and efficiency. The key to this effect is regulating the environmental humidity during solvent evaporation, which presumably enables spatiotemporal modulation of the polymerization and crystallization processes. Such control over the film structure allows statistical evaluation of morphology‐dependent variations in chemiresistive response, demonstrating that this operationally simple approach provides a robust platform for both fundamental studies and technological development of COF films.

## Graphical abstract

We develop an operationally simple, efficient approach to prepare thin films of 2D imine COFs, where environmental humidity plays a key role in the formation of smooth, face‐on oriented films. The high controllability and reproducibility of this method allow statistical evaluation of morphologically varied COF films for chemiresistive sensing, demonstrating that uniformity in film structure is critical for consistent performance.

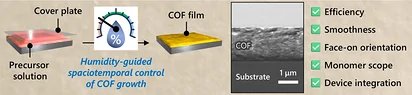

## 1 Introduction

Fabrication of high‐quality thin films with precise morphological control is a key challenge in materials science. Indeed, the morphology and molecular organization of functional active layers can be just as important as chemical composition and molecular structure in determining device performance. This consideration is especially critical for organic semiconductors, in which subtle variations in film structure can strongly influence electric properties and device characteristics. Accordingly, substantial research efforts have been devoted to developing reliable strategies for controlling the structure of organic semiconductor films [1, 2, 3, 4, 5, 6].

Covalent organic frameworks (COFs) are a class of porous crystalline polymers featuring extended two‐ or three‐dimensional covalent networks, and have emerged as promising materials for diverse applications, including gas separation, molecular sieving, catalysis, and electronics [7, 8, 9, 10, 11, 12, 13, 14, 15, 16, 17]. Many of these applications require a smooth and homogeneous active layer; consequently, the controlled preparation of COF films has become increasingly important as researchers continue to explore the potential of this class of materials [14, 18, 19, 20, 21, 22, 23]. In general, preparing COF films through post‐synthetic processing (i.e., the “top‐down strategy”) is challenging because COFs are typically obtained as insoluble microcrystalline powders, although several successful examples have been reported [24, 25, 26, 27, 28, 29, 30, 31, 32]. On the other hand, the “bottom‐up strategy,” in which COFs are synthesized directly in the form of thin films, has been more widely employed.

The most common and seemingly simplest bottom‐up approach involves placing a substrate in a reaction mixture during the conventional solvothermal COF synthesis [33, 34, 35, 36, 37, 38, 39, 40, 41, 42, 43, 44]. However, the efficiency of this approach is low, as the majority of the product precipitates at uncontrolled locations within the reaction vessel. Moreover, the outcome is sensitive to setup‐specific factors such as the shape of the reaction vessel and the position or orientation of the substrate, often resulting in limited reproducibility. Another commonly used approach is the synthesis of COFs at a liquid/liquid or gas/liquid interface. Such interfacial syntheses have successfully produced high‐quality, large‐area thin films [45, 46, 47, 48, 49, 50, 51, 52, 53, 54, 55]. Nonetheless, their efficiency is also often limited in terms of material yield and processing time. In addition, films obtained via this approach can be prone to cracking and buckling during transfer to solid substrates for device integration.

Given these challenges, we are developing an efficient and broadly applicable approach for preparing high‐quality COF films. Herein, we report a simple yet effective in situ polymerization method, in which a monomer‐containing solution is drop‐cast onto a substrate and subsequently evaporated under controlled conditions to promote COF formation. Drop‐casting‐based processes are attractive because they offer high efficiency in both material usage (via confined polymerization within the drop‐cast area) and processing time (typically requiring a few hours at most). This advantage has been demonstrated by several groups; for example, Liu and coworkers obtained homogeneous COF films in approximately 10 min [56]. More recently, Li et al. reported a “modulator and solvent‐induced polymerization” method, in which a precursor solution was dried in 15 min to yield a COF film on a substrate [57]. However, the drop‐casting process is subtle and complex [58, 59], and the quality of the resulting films is often affected by non‐obvious factors. Our method addresses this issue through careful optimization of evaporation conditions—in particular, the relative humidity (RH) of the surrounding atmosphere. This approach enables the reliable formation of smooth films and, at the same time, promotes a high degree of face‐on orientation of COF crystallites. In the following sections, we will detail this method and demonstrate that such structural control is invaluable for developing COF films as device active layers.

## 2 Results and Discussion

### 2.1 Key Design of the In Situ Polymerization Method

**FIGURE 1:**
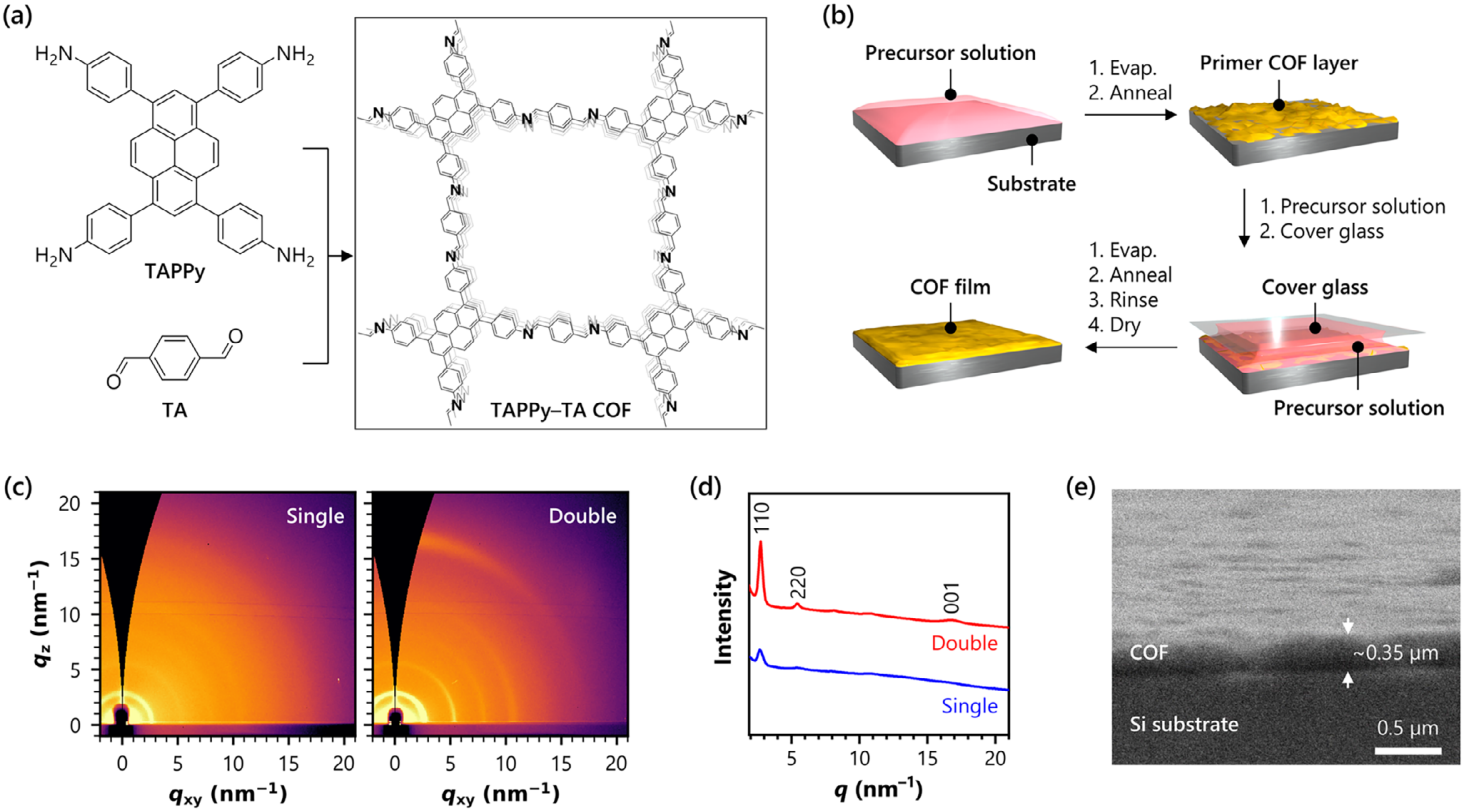
Development and preliminary validation of the method. (a) Chemical structures of TAPPy–TA COF and the corresponding monomers, TAPPy and TA. (b) Schematic illustration of the drop‐casting‐based method developed in this work. (c) 2D GIWAXS patterns of TAPPy–TA COF films prepared by single (left) and double (right) drop casting. The volumes of the cast precursor solution are 50 µL for single drop casting and 2 × 20 µL for double drop casting. (d) Azimuthally integrated intensity profiles derived from the 2D GIWAXS data in panel c. (e) Cross‐sectional scanning electron microscope (SEM) image of a TAPPy–TA COF film prepared by double drop casting on a silicon substrate.

An imine COF constructed from 1,3,6,8‐tetrakis(4‐aminophenyl)pyrene (TAPPy) as the node and terephthalaldehyde (TA) as the linker, denoted here as TAPPy–TA COF, was selected as a model system (Figure 1a). This two‐dimensional (2D) COF is characterized by high crystallinity and stability, resulting from the strong stacking between *π*‐conjugated layers, and holds promise for electronic and optoelectronic applications [60]. At the same time, the strong tendency of TAPPy to aggregate can hinder the controlled deposition of the resulting polymer; thus, achieving high‐quality films of this COF would represent a significant step toward establishing a robust and broadly applicable method for preparing COF films.

The precursor solution for in situ polymerization was prepared by dissolving the monomers in a stoichiometric molar ratio of TAPPy:TA = 1:2 in a mixture of benzonitrile/1,4‐dioxane/trifluoroacetic acid (TFA)/water (70:30:20:1, v/v; Table S1). A small amount of water was added to retard imine condensation by shifting the reaction equilibrium toward the monomer side. TFA serves not only as a catalyst for imine formation but also as a suppressor of oligomer aggregation. The suppressing effect originates from protonation of imine moieties, which induces electrostatic repulsion among the growing oligomers. A related strategy has been employed for post‐synthetic delamination of 2D imine COFs to eventually deposit them via a top‐down approach [27]. Omitting water or replacing TFA with weaker acids, such as acetic acid or formic acid, resulted in rapid precipitation, preventing the formation of smooth COF films (Figure S4). Benzonitrile and 1,4‐dioxane were selected as the primary solvents because they readily dissolve the monomers and exhibit evaporation rates suitable for drop casting—sufficiently fast for efficient film formation yet slow enough for fabricating crystalline frameworks. In contrast, replacing benzonitrile with acetonitrile resulted in a narrower processing window, most likely owing to its higher volatility and lower solubilizing capability toward the monomers or intermediate species. Upon preparation, the precursor solution immediately changed color from light yellow to orange‐red, indicating partial imine formation already at this stage (Figures S4–S6) [61].

The solution was then drop‐cast onto a substrate and allowed to evaporate under gentle heating at 60°C over several minutes (Figure 1b and Figure S7). Either glass or silicon substrates were used; for silicon, optional pretreatment with (3‐aminopropyl)triethoxysilane improved film adhesion. This simple drop‐casting procedure yielded the expected COF, as confirmed by 2D grazing‐incidence wide‐angle x‐ray scattering (GIWAXS). Specifically, the resulting film exhibited a scattering arc at *q* = 2.7 nm^−1^ (*d* = 2.3 nm), corresponding to the 110 reflection of the TAPPy–TA COF (Figure 1c,d) [60]. The rapid formation of the crystalline framework is consistent with previous reports showing that 2D imine COFs can form within several minutes [62, 63].

Further optimization of the process revealed that repeated drop casting markedly improves film quality. In this approach, one or more additional solution‐deposition–evaporation cycles are performed after formation of the initial layer. The optimized protocol also involves covering the deposited solution with a glass plate during solvent evaporation in the final casting step (Figure 1b). When applied in the initial step, the cover glass often caused the solution to spill over the substrate; thus, a pre‐formed layer is required as a primer that helps retain the solution during the subsequent casting step using the cover glass. Comparison of the 2D GIWAXS patterns shows distinctly stronger and sharper scattering peaks for a film prepared by double drop casting than for one prepared by single casting (Figure 1c,d). This enhanced crystallinity likely arose from prolonged solvent evaporation, which facilitated error correction in covalent networks via dynamic bond exchange (Figure S8a,b). On the other hand, the surface morphology is largely unaffected by the use of a cover glass (Figure S8c). Notably, sharp x‐ray scattering peaks were observed even with a small number of drop‐casting cycles when a cover glass was applied during the final casting step, indicating that the cover‐glass treatment plays a dominant role in improving the crystallinity of the resulting COF films (Figure S9).

Based on these results, we established the key design of our bottom‐up approach, which involves multiple drop‐casting cycles with a cover glass applied in the final evaporation step. This straightforward approach yields continuous films having smooth surfaces and uniform thickness (Figure 1e). In addition, the overall deposition process takes approximately 1–2 h, which is significantly shorter than most reported methods for preparation of COF films [18]. Indeed, this duration is comparable to that required for fabricating high‐quality films of molecular semiconductors via carefully controlled solution‐based techniques [64]. However, subsequent experiments revealed limited reproducibility—the smoothness and homogeneity of the films varied with season. Specifically, smooth films were obtained most reliably in winter at the experimental site (Osaka, Japan), whereas those prepared in summer frequently exhibited highly rough surfaces. Our efforts to address this issue are described in the following sections.

### 2.2 Effect of Ambient Humidity on Film Structure

**FIGURE 2:**
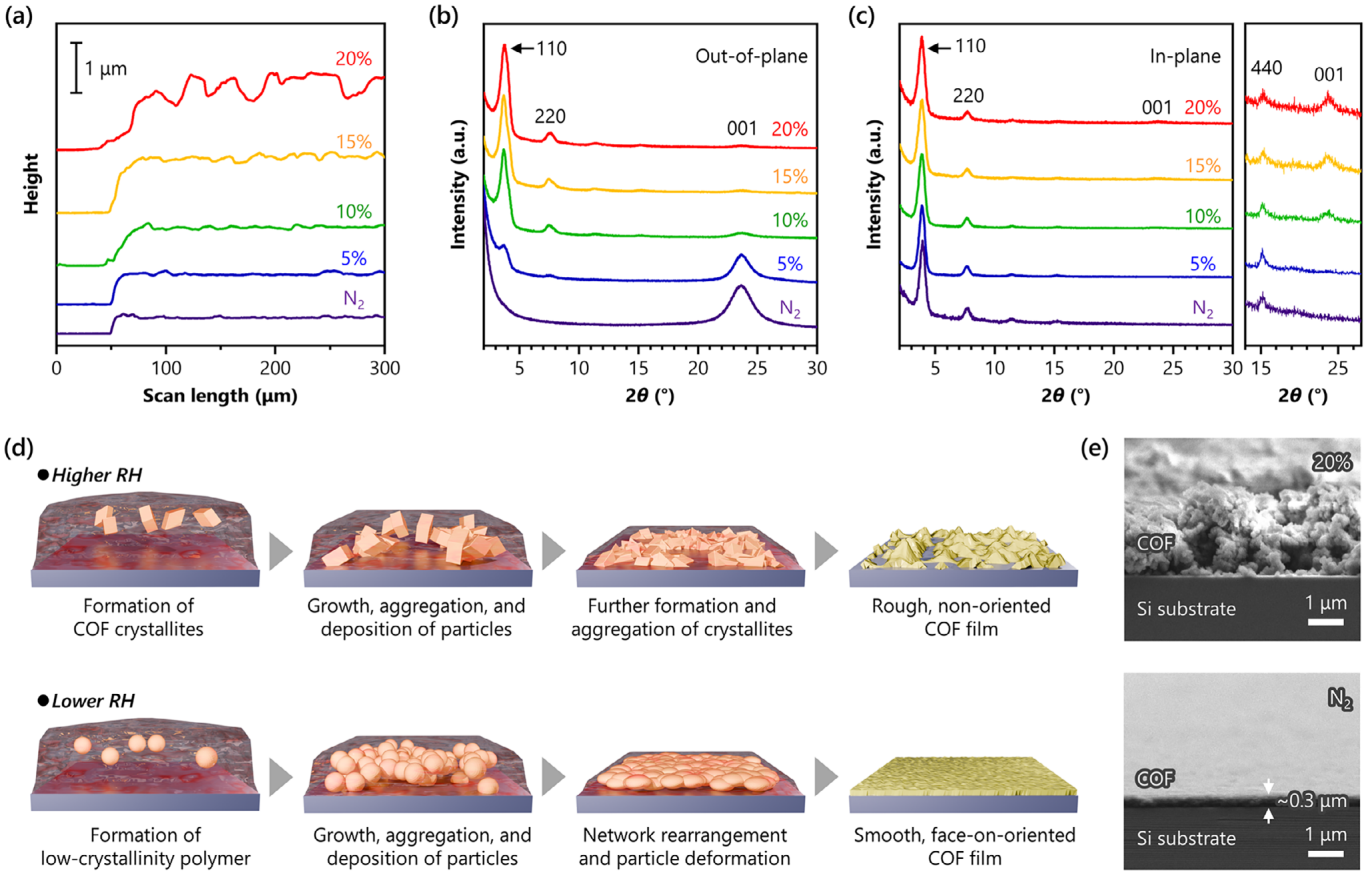
Influence of ambient humidity during in situ polymerization on the structural evolution of TAPPy–TA COF films. (a) Surface profiles of films prepared by double drop casting at varying RH. (b, c) Out‐of‐plane and in‐plane XRD patterns of films prepared by double drop casting at different RH. The in‐plane XRD patterns are vertically expanded to highlight the presence or absence of the 001 reflection. (d) Proposed formation pathways of COF films under higher (top) and lower (bottom) RH conditions. (e) Cross‐sectional SEM images of films prepared by double drop casting at 20% RH (top) and under N_2_ (bottom).

The seasonal variation in film morphology suggested that ambient conditions significantly influence the film‐formation process. Systematic screening of deposition conditions revealed that ambient RH has a substantial influence on the formation of COF films. Stylus profiler analysis showed a clear trend: higher RH led to progressively rougher surfaces, whereas highly smooth films were obtained only when the RH was approximately 10% or lower (Figure 2a). This result is consistent with the earlier observation that smooth films formed most reproducibly during the driest season at our experimental site (i.e., in winter). Films prepared under low‐humidity conditions exhibited not only smoother surfaces but also a stronger preference for the face‐on crystallite orientation, in which the 2D COF sheets align parallel to the substrate. Under extremely dry conditions (dry N_2_, as a substitute for 0% RH in air), a pronounced face‐on orientation was achieved. X‐ray diffraction (XRD) analyses of the resulting film showed diffraction peaks from the (001) and (110) planes exclusively in the out‐of‐plane and in‐plane modes, respectively (Figure 2b,c). This trend was further corroborated by 2D GIWAXS data and the corresponding Hermans orientation factors (Figures S10 and S11). It should be also noted that variations in the water content of the precursor solution have a far smaller effect on the resulting film morphology than ambient RH (Figure S12).

**FIGURE 3:**
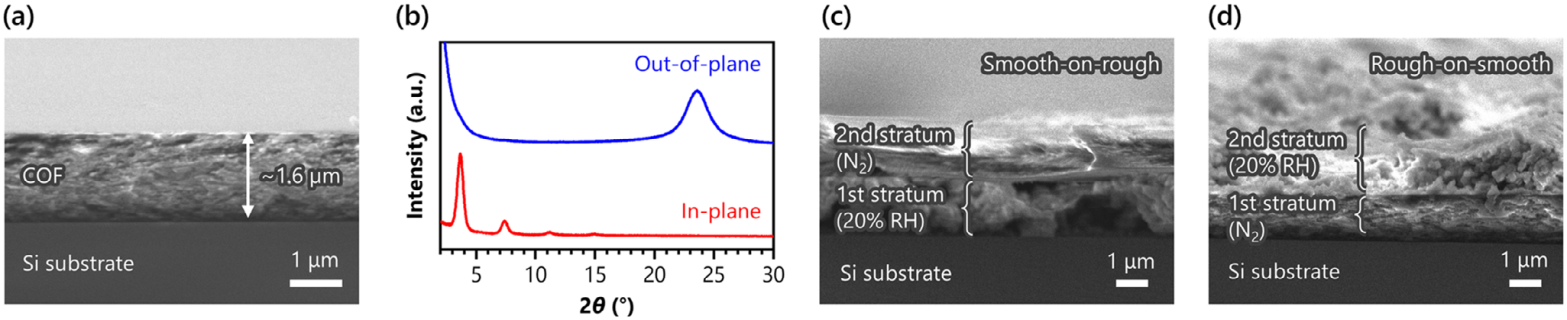
Engineering of film architecture under controlled humidity. (a) Cross‐sectional SEM image of a TAPPy–TA COF film prepared by 10 drop‐casting cycles. (b) Out‐of‐plane and in‐plane XRD patterns of the same film shown in panel a. (c, d) Cross‐sectional SEM images of “smooth‐on‐rough” and “rough‐on‐smooth” films prepared by 5 drop‐casting cycles at 20% RH followed by 5 cycles under N_2_, or in the reverse order.

We postulate that the strong dependence of film structure on RH arises from changes in the spatiotemporal profile of crystallization during solvent evaporation (Figure 2d). Under low‐RH conditions, the water content in the drying solution should be lower than under high RH, which reduces the reversibility of imine‐bond formation. Solvent evaporation under these conditions would favor early‐stage formation of low‐crystallinity or amorphous polymer networks. Crystallization into the COF would then occur primarily at the later stages of solvent loss within a concentrated, confined thin liquid (or gel‐like) film near the substrate. The structural flexibility of such a later‐stage film, combined with still‐operative dynamic covalent exchange, may allow the material to form a smooth, continuous film without noticeable cracking or warping. At the same time, morphological relaxation and bond rearrangement within this confined volume likely promote preferential formation of face‐on‐oriented COF films, presumably driven by macroscopic factors such as mechanical‐stress relaxation and entropy‐driven ordering [65, 66]. This interpretation is in line with a recent study by Cusin and coworkers, in which the authors attributed face‐on orientation in 2D COF films to the relaxation of tensile stress that counteracts the compressive stress imposed on flat, inert substrates during solvent evaporation [67]. Although the details of our system may differ—flat surface is not a prerequisite in our case as described below (Figure 3c)—both processes seem to share certain features, including early formation of low‐crystallinity networks and subsequent evolution toward strong face‐on alignment through structural adaptation during later stages of solvent evaporation (Figure 2d).

At higher RH, the increased water content in the deposited solution is expected to ensure effective reversibility of imine formation, promoting a more controlled polymerization process. Consequently, COF crystallites are likely to nucleate earlier than those formed under lower RH conditions, with subsequent growth and aggregation occurring in the bulk liquid phase. Continued solvent evaporation would further drive this aggregation, ultimately yielding large clusters. Capillary forces among these particles may additionally reinforce this consolidation during the final stages of drying. Such randomly and densely packed crystallite assemblies should be difficult to reorganize in a way that allows a preferred orientation to develop. Moreover, aggregated crystallites should be far less deformable than the amorphous or low‐crystallinity polymer networks formed under lower RH conditions, thereby hindering structural adaptation and leading to rough surfaces. Consistent with this scenario, SEM and atomic force microscope (AFM) images show markedly larger aggregates in films prepared at higher RH, supporting enhanced crystallite aggregation under these conditions (Figure 2e and Figure S13).

### 2.3 Humidity‐Guided Engineering of Film Architecture

The humidity‐controlled approach enables robust and reproducible regulation over the architecture of resulting COF films. By repeating the drop‐casting sequence, the film thickness can be tuned over a wide range; in this study, thicknesses from approximately 0.3 µm to beyond 1.6 µm were achieved (Figures 2e and 3a). Notably, deposition under an N_2_ atmosphere preserves the face‐on crystallite orientation even in micrometer‐thick films (Figure 3b). This behavior contrasts sharply with conventional solution processing of crystalline organic materials, in which substrate or interfacial templating is typically effective only in very thin films, generally limited to a few tens of nanometers [68, 69, 70, 71]. Importantly, surface smoothness is maintained across the entire thickness range, as confirmed by SEM and AFM measurements (Figure S14). Whereas increasing thickness is commonly accompanied by crack formation or surface roughening, no discernible thickness‐dependent morphological degradation was observed in COF films prepared by the present method. These results support the proposed film‐formation mechanism, in which the face‐on orientation and smooth surface are governed not by interfacial templating but by macroscopic effects operative while the film remains deformable and capable of accommodating drying‐induced mechanical stresses.

Beyond thickness control, the method enables deliberate construction of stratified architectures with distinct structural characteristics. A “smooth‐on‐rough” architecture was realized by depositing the first layer at 20% RH, followed by the second layer under N_2_. The cross‐sectional SEM image of the resulting sample clearly resolves two morphologically distinct strata, while the out‐of‐plane XRD pattern appears as a superposition of face‐on‐oriented and non‐oriented profiles (Figure 3c and Figure S15a). This observation further supports the insignificant role of substrate templating in the formation of face‐on‐selective smooth COF films via the present approach.

Conversely, a “rough‐on‐smooth” architecture can be obtained by reversing the order of deposition sequence; namely, first under N_2_ and subsequently at 20% RH. Distinct morphological strata are again evident in the cross‐sectional SEM image, and the corresponding XRD pattern is likewise consistent with a superposition of the face‐on‐oriented and non‐oriented components (Figure 3d and Figure S15b). The structural versatility achieved by the sequential deposition at varied humidity levels may be advantageous for the construction of hierarchically porous COF films that integrate, for example, molecular sieving with catalytic or sensing functionalities [72, 73].

### 2.4 Pore Accessibility in Face‐on‐Oriented COF Films

Face‐on‐oriented COF films on substrates are particularly attractive for technological applications, as this orientation facilitates efficient guest uptake and diffusion in intrinsic pores of 2D COF crystallites. To assess pore accessibility in COF films prepared under an N_2_ atmosphere, we examined their response to moisture in the film form and conducted gas sorption analysis on a powder sample collected from multiple films.

**FIGURE 4:**
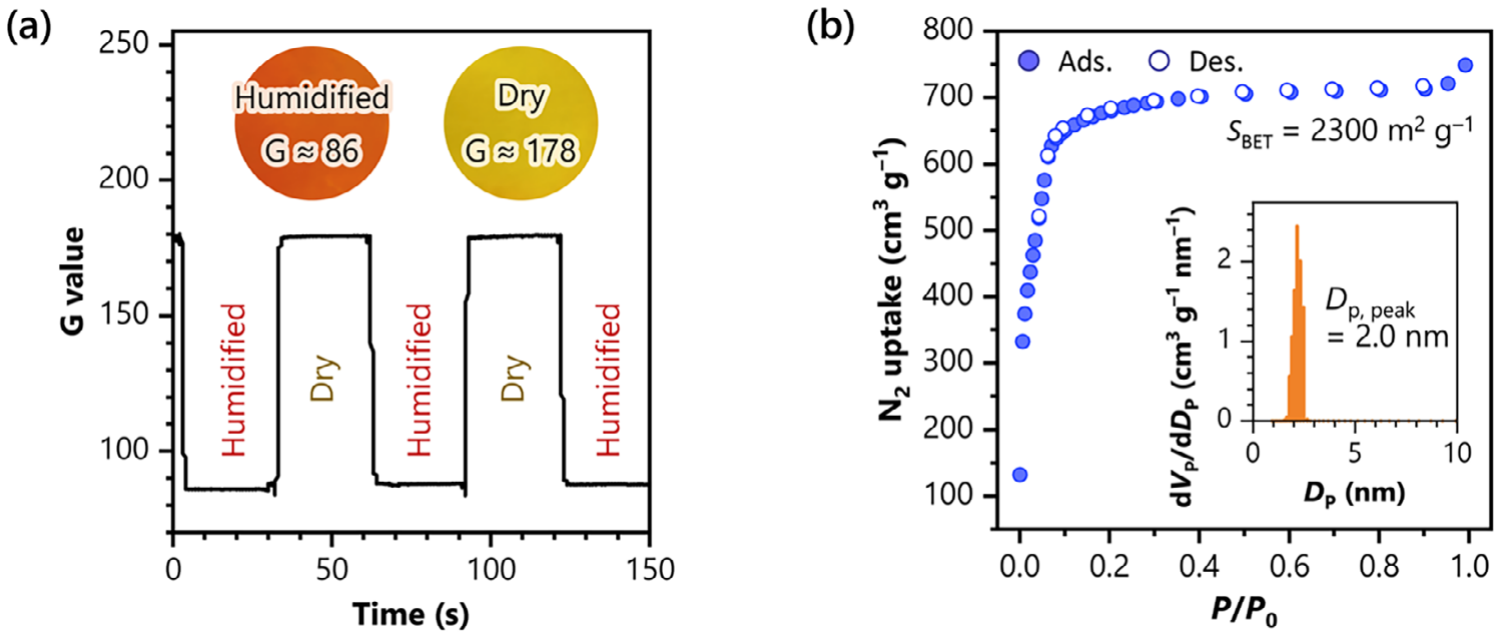
Porosity evaluation of face‐on oriented TAPPy–TA COF films. (a) Change in apparent color upon exposure to moisture. The color information was extracted in RGB format, and the plot shows the G value. The film was prepared by triple drop casting on glass. (b) N_2_ sorption data measured at 77 K and estimated pore size distribution (inset) of a powder sample collected from multiple films.

TAPPy–TA COF is known to exhibit solvatochromism to moisture, characterized by a distinct color change from yellow to red [74]. This phenomenon has been attributed to electronic perturbations induced by the incorporation of guest water molecules and serves as a convenient indicator of pore accessibility. Consistent with the previous report, films prepared under N_2_ exhibited a rapid and reversible color change upon exposure to moisture (Figure 4a). In contrast, amorphous films prepared from TAPPy and TA did not show distinctive color change under identical conditions, underscoring the critical role of a crystalline porous framework in enabling the solvatochromic response (Figure S16).

The porosity of the films was further validated by N_2_ sorption analysis at 77 K. The Brunauer–Emmett–Teller surface area (*S*_BET_) was determined to be 2300 m^2^ g^−1^, and nonlocal density functional theory (NLDFT) analysis revealed a narrow pore size distribution centered at 2.0 nm (Figure 4b). These values are comparable to those previously reported for TAPPy–TA COF [33, 74]. Although the collected powder sample may have undergone minor structural change during scraping (Figure S17), the high surface area and well‐defined pore size distribution nonetheless confirm that the COF films prepared by the present method retain high pore accessibility.

### 2.5 Relationship Between Film Structure and Chemiresistive Response to Moisture

**FIGURE 5:**
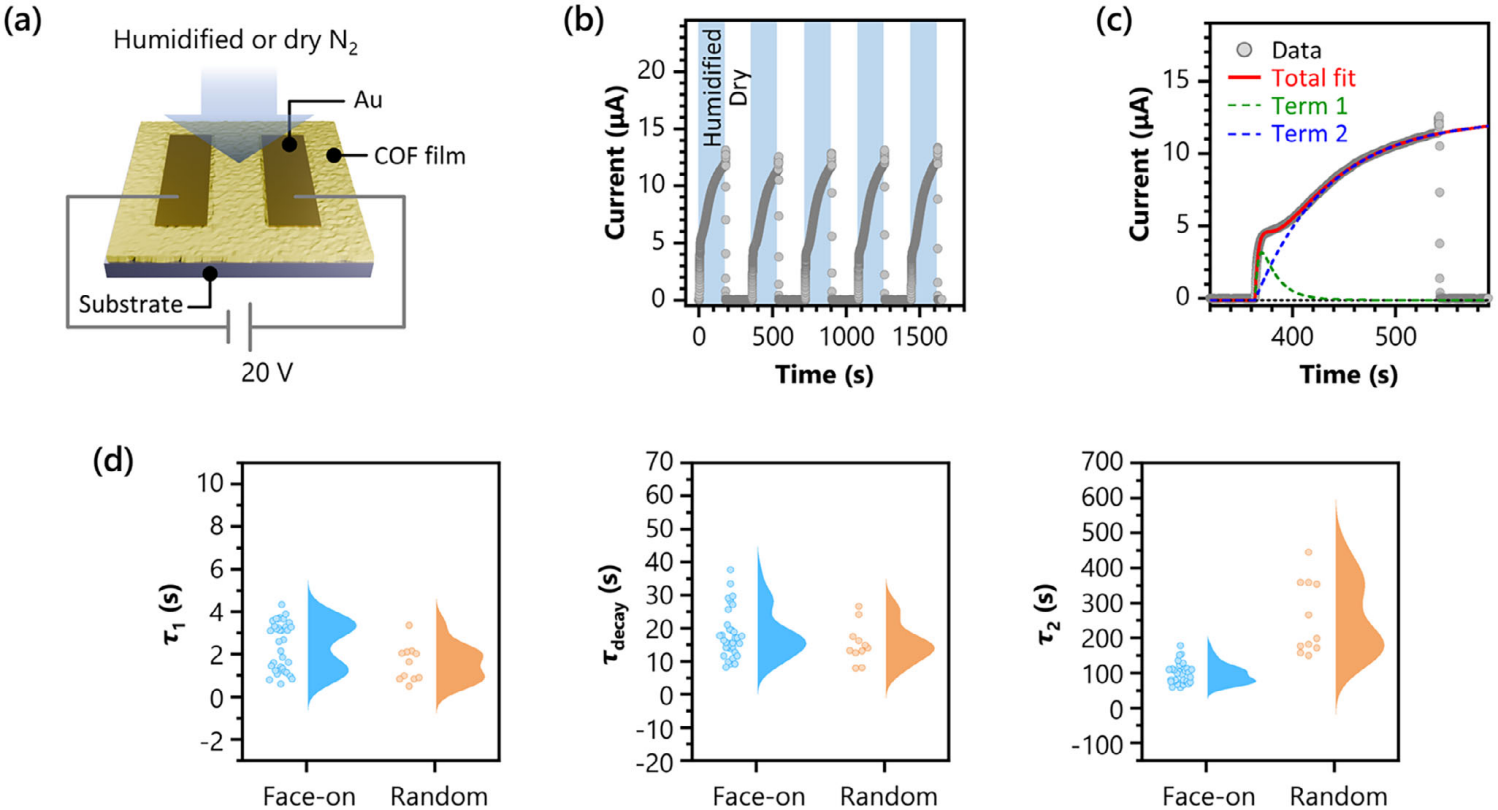
Chemiresistive response of TAPPy–TA COF films to moisture. (a) Schematic illustration of the measurement setup. (b) Change in electric current for a face‐on‐oriented film at an applied bias of 20 V. Channel length: 0.05 mm, channel width: 5 mm. (c) Representative curve fitting of an “on” segment in panel b. Fitting results: *τ*_1_ = 4 s, *τ*_decay_ = 15 s, *τ*_2_ = 72 s, R^2^ = 0.9996. (d) Violin plots to compare the distributions of time constants between face‐on‐oriented and non‐oriented (random) films. The datasets comprise 35 and 11 points for face‐on and random films, respectively. All films were prepared on glass substrates by triple drop casting.

Imine‐linked COFs exhibit chemiresistive responses to moisture, characterized by on/off modulation of the electric current under an applied bias. In particular, a COF comprising 1,3,5‐tris(4‐aminophenyl)benzene (TAPB) and TA shows a substantial increase in current upon exposure to moisture at 20 V [75]. Building on this precedent, we examined the chemiresistive response of TAPPy–TA films with different structures. Because the chemiresistive signal reflects charge transport across the entire channel between electrodes, it can serve as a probe of the macroscopic film structure. Herein, we focus on two representative film types: highly smooth, face‐on‐oriented films prepared under N_2_, and slightly rough, non‐oriented (random) films prepared at 10% RH. Devices were fabricated by depositing gold electrodes onto each COF film, yielding a channel length of 0.05 mm and a channel width of 5 mm. The electrical response was measured under a constant bias of 20 V (Figure 5a).

Both face‐on‐oriented and non‐oriented films exhibited pronounced chemiresistive responses to moisture (Figure 5b, Figure S18a). Under dry N_2_ flow, the current was negligible (∼10^−9^ A) for both film types. Upon switching the gas flow to humidified N_2_, the current increased to the order of 10^−6^ A or higher, corresponding to on/off ratios exceeding 10^3^. Reintroduction of dry N_2_ resulted in rapid reduction of the current to its initial low level within a few seconds, demonstrating fast and reversible switching.

Analysis of the transient current profiles revealed a two‐stage increase consisting of a rapid initial rise followed by a slower secondary growth. Fitting these profiles with a multi‐component kinetic model yielded three characteristic time constants, *τ*_1_, *τ*_decay_, and *τ*_2_ (Figure 5c and Figure S18b; for details). Although the precise physical origins remain to be clarified, *τ*_1_ and *τ*_decay_ are tentatively attributed to surface or interface phenomena, whereas subsequent molecular or framework reorganization may explain *τ*_2_. In relation to this interpretation, GIWAXS measurements have revealed a subtle decrease in interlayer spacing between stacked COF sheets under high‐RH conditions (Figure S19).

In addition, the extracted time constants were statistically compared between the two film types with the aim of probing the impact of film structure on response reproducibility (Figure 5d, Table S2). The distributions of *τ*_1_ and *τ*_decay_ are similar between face‐on‐oriented and non‐oriented films. In contrast, *τ*_2_ is larger on average and exhibits a substantially broader distribution for the non‐oriented films. The markedly higher consistency of *τ*_2_ observed for the highly smooth, face‐on‐oriented films demonstrates that a well‐defined, homogeneous structure plays a critical role in facilitating reproducible dynamic charge transport. These results highlight the importance of precise morphological control for practical applications of COF films, particularly in sensing devices where response consistency is critical.

### 2.6 Generality of the Humidity‐Controlled Approach

The generality of the humidity‐controlled drop casting for preparing smooth, face‐on‐oriented COF films was evaluated by extending the protocol to different monomer combinations. In each case, the composition of the precursor solution was individually optimized to suppress premature polymer precipitation prior to drop casting and to ensure reproducible formation of crystalline films (Table S1).

**FIGURE 6:**
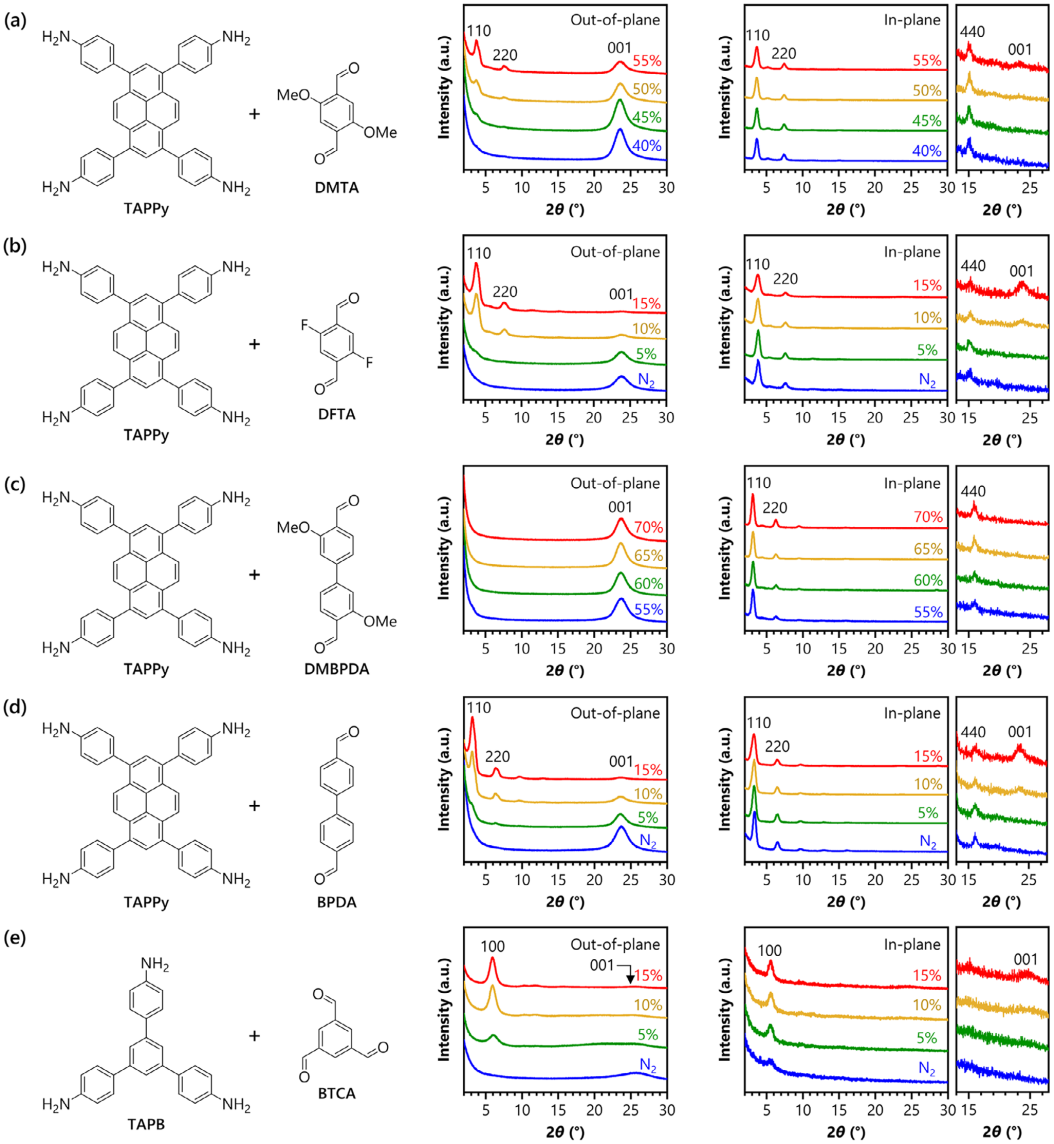
Generality of the humidity‐controlled film preparation across imine COFs. In each panel, the chemical structures of the monomers and the out‐of‐plane and in‐plane XRD patterns of COF films prepared at different RH are shown. The in‐plane XRD patterns are vertically expanded to highlight the presence or absence of the 001 reflection. The films were prepared on silicon substrates by double drop casting for panels a–d and triple drop casting for panel e. (a) TAPPy–DMTA COF. (b) TAPPy–DFTA COF. (c) TAPPy–DMBPDA COF. (d) TAPPy–BPDA COF. (e) TAPB–BTCA COF.

We first prepared TAPPy‐based COFs using a series of dialdehyde linkers other than TA, namely 2,5‐dimethoxyterephthalaldehyde (DMTA), 2,5‐difluoroterephthalaldehyde (DFTA), 3,3ʹ‐dimethoxy‐[1,1ʹ‐biphenyl]‐4,4ʹ‐dicarbaldehyde (DMBPDA), and [1,1ʹ‐biphenyl]‐4,4ʹ‐dicarbaldehyde (BPDA). TAPPy–DMTA [76] and TAPPy–DFTA [76] were deposited under varied RH, and XRD analysis revealed strong face‐on orientation at RH values below approximately 40% and 5%, respectively (Figure 6a,b). SEM and AFM analyses confirmed the formation of smooth films under these conditions (Figure S20). Notably, Cusin and coworkers have reported that DMTA undergoes acid‐promoted imine condensation significantly faster than both TA and DFTA [67]. They proposed that the accelerated condensation favors kinetically controlled polymerization, leading to the formation of amorphous three‐dimensional networks that transform into face‐on‐aligned networks under tensile stresses induced by solvent evaporation. Consistent with this report, DMTA promoted face‐on orientation more readily than TA and DFTA, yielding face‐on‐oriented films over a broader RH range. This observation further supports the mechanism proposed herein for humidity‐controlled film formation, in which the early‐stage generation of low‐crystallinity polymeric species during solvent evaporation plays a key role in determining film structure. Additionally, the TAPPy–DMTA system forms amorphous films upon single drop casting at 40% RH (Figure S21), also consistent with the proposed evolution from an amorphous intermediate to a crystalline COF during the later stages of the deposition process.

The applicability of the method was further examined using the biphenyl‐based linkers DMBPDA and BPDA. (Figure 6c), which is the upper limit of our experimental setup (Figure S1). Note that the TAPPy–DMBPDA formed an amorphous film when deposited by single‐drop casting at this high humidity level (Figure S21). Thus, here again, it would be reasonable to assume that the formation of low‐crystallinity intermediates is associated with the generation of high‐quality COF films. In contrast, TAPPy–BPDA exhibited a strong tendency to form low‐crystallinity films. This issue was mitigated by introducing aniline as a modulator, enabling reproducible formation of smooth, oriented films at RH values below approximately 5% (Figure 6d and Figure S20). Note that TAPPy–BPDA has previously been reported to lose crystallinity upon solvent evaporation [60, 77]. Nevertheless, crystalline films of this COF were obtained via the same polymerization and post‐synthetic treatment conditions as those used for the other COFs, indicating the robustness of the present procedure.

Finally, the method was extended to a COF with a different framework topology, constructed from TAPB and benzene‐1,3,5‐tricarbaldehyde (BTCA) [78]. In this case, a highly selective face‐on orientation of the crystallites was achieved only under an N_2_ atmosphere. It is notable that the intensity of the 100 reflection in the in‐plane XRD pattern of the face‐on‐oriented film is weaker than that observed for films prepared at 5% RH or higher (Figure 6e). This result suggests that the drop‐casting conditions have not been fully optimized; nevertheless, the RH dependence of crystallite orientation, along with the high surface smoothness of the face‐on‐oriented film (Figure S20), is evident.

Furthermore, films of these five 2D imine COFs all showed porosity characteristics comparable to those of their bulk counterparts (Figure S22). Collectively, these results demonstrate that the humidity‐controlled drop‐casting strategy is broadly applicable to diverse imine COFs, while also revealing that monomer reactivity and framework formation kinetics define the humidity window required to achieve smooth, face‐on‐oriented films.

## 3 Conclusion

In polymerizations via reversible dehydration condensation, ambient humidity is inherently expected to influence reaction kinetics and polymer growth. Despite this fundamental relevance, limited attention has been paid to the role of humidity in the bottom‐up fabrication of COF films. In this study, we identified humidity as a critical parameter to achieve highly smooth, face‐on‐oriented films of 2D imine COFs in a drop‐casting‐based in situ polymerization approach. Our results indicate that variations in RH would modulate the spatiotemporal evolution of polymer networks, determining whether low‐crystallinity intermediates or highly crystalline particles predominate at different stages of solvent evaporation.

This simple, efficient humidity‐controlled approach is expected to be valuable for the development of COF films for those applications where structural order, morphological uniformity, and pore accessibility are essential. Indeed, a statistical evaluation of TAPPy–TA COF films showed that variations in film structure markedly influence the reproducibility of their chemiresistive response to moisture. We also demonstrated the applicability of the present approach to a range of monomers with varying reactivities, sizes, and topologies.

Taken together, the combination of reliable structural control and broad monomer scope establishes the present approach as a robust platform for the preparation of 2D imine‐linked COF films. While extending this strategy to other classes of COFs, including 3D frameworks, remains an important direction for future research, the present work opens new opportunities for fully exploiting COFs as active components in thin‐film devices, as well as for investigating the interplay among chemical structure, film morphology, and device performance.

The authors have cited additional references within the Supporting Information [79, 80, 81, 82].

## Conflicts of Interest

The authors declare no conflicts of interest.

## Supporting information




**Supporting File**: smll74867‐sup‐0001‐SuppMat.pdf.

## Data Availability

The data that support the findings of this study are available from the corresponding author upon reasonable request.
