# Supplementary material for: Humidity‐Guided In Situ Polymerization for Controlled Formation of Smooth, Oriented Covalent Organic Framework Films
Source: Small. 2026 Jul 31;22(54):e74867. doi: 10.1002/smll.74867 (PMC13614274; doi:10.1002/smll.74867)
Supplement: Supplementary file 1 [file SMLL-22-e74867-s001.pdf]

Supporting Information for

Humidity-Guided In Situ Polymerization for Controlled Formation of Smooth, Oriented Covalent Organic Framework Films

Mitsuharu Suzuki,[1,*] Sora Yamazaki,[1] Yoshiki Shirota,[1]
Satoshi Haze,[1] Junya Yasaka,[1] Ryunosuke Sakamoto,[2] Naoya Aizawa,[1,3] Osamu Ikeda,[4]
Tomoyuki Koganezawa,[4] Rosantha Kumara,[4] and Ken-ichi Nakayama[1]

[1]*Division of Applied Chemistry, Graduate School of Engineering, The University of Osaka, 2-1 Yamadaoka, Suita, Osaka 565-0871, Japan.*

[2]*Department of Applied Chemistry, Division of Applied Science, School of Engineering, The University of Osaka, 2-1 Yamadaoka, Suita, Osaka 565-0871, Japan*

[3]*Institute for Chemical Reaction Design and Discovery (WPI-ICReDD), Hokkaido University, Kita 21 Nishi 10, Kita-ku, Sapporo, Hokkaido 001-0021, Japan.*

[4]*Japan Synchrotron Radiation Research Institute (JASRI), 1-1-1 Kouto, Sayo-cho, Sayo-gun, Hyogo 679-5198, Japan.*

Correspondence should be addressed to
M.S. (msuzuki@chem.eng.osaka-u.ac.jp)

Contents

S1. Experimental details 2

S1.1. General information 2

S1.2. In situ polymerization for film preparation 3

S1.3. Evaluation of moisture response of TAPPy–TA COF films 4

S1.4. Solvothermal synthesis and structural characterization of TAPPy–DMBPDA COF 5

S2. Supplementary data 7

S2.1. Development of the film formation method 7

S2.2. GIWAXS analysis of TAPPy–TA COF films prepared at different humidity levels 10

S2.3. Effect of water content in the precursor solution 12

S2.4. SEM and AFM characterization of TAPPy–TA COF films prepared at different humidity levels 12

S2.5. Structural characterization of humidity-guided architectures of TAPPy–TA COF films 13

S2.6. Solvatochromic response of amorphous TAPPy–TA films to moisture 14

S2.7. XRD analysis of TAPPy–TA COF powder collected from face-on oriented films 15

S2.8. Chemiresistive response of TAPPy–TA COF films to moisture 16

S2.9. GIWAXS analysis of a face-on oriented TAPPy–TA COF film under controlled humidity 18

S2.10. SEM and AFM characterization of face-on-oriented COF films other than TAPPy–TA 18

S2.11. Formation of low-crystallinity films with DMTA or DMBPDA 20

S2.12. Porosity analysis of COF films other than TAPPy–TA 20

Supplementary references 22

S1. Experimental details

S1.1. General information

All solvents and reagents were purchased from commercial suppliers and used as received unless otherwise noted.

One-dimensional X-ray diffraction (XRD) patterns of film samples were recorded on a SmartLab X-ray diffractometer (Rigaku) equipped with a D/teX Ultra 1D silicon strip detector and a Cu-Kα radiation source operated at 9 kW. The measurements were performed in the parallel-beam geometry over a scan range of 2θ = 2–30° at a scan rate of 1.2° min^{-1}. For qualitative XRD assessment of film and powder samples, diffraction patterns were alternatively acquired using a MiniFlex600 desktop X-ray diffractometer (Rigaku) equipped with a D/teX Ultra 1D silicon strip detector and a Cu-Kα radiation source operated at 600 W, over a scan range of 2θ = 2–30° at a scan rate of 2° min^{-1}.

Two-dimensional grazing-incidence wide-angle X-ray scattering (2D GIWAXS) measurements were conducted at beamline BL19B2 of SPring-8. The incident X-ray beam was monochromatized with a double-crystal Si(111) monochromator to an energy of 12.4 keV (λ = 0.100 nm). The incident angle was fixed at 0.12° using a Huber diffractometer, and the sample-to-detector distance was approximately 175.7 mm. Scattered X-rays were recorded for 30 s using a PILATUS 300K X-ray photon counting pixel detector (DECTRIS).

Film thickness and surface topology profiles were measured with a DektakXT surface profiler (Bruker). Scanning electron microscopy (SEM) images were obtained using a JSM-F100 microscope (JEOL) operated at an accelerating voltage of 3 kV under a pressure below 10^{-3} Pa. Atomic force microscopy (AFM) measurements were performed using an SPM-9700HT microscope (Shimazu) in the dynamic mode.

N_2 sorption isotherms were measured at 77 K using a BELSORP MINI surface-area and pore-size-distribution analyzer (MICROTRAC). Prior to measurement, samples were vacuum-dried to remove residual solvents using a BELPREP VAC II vacuum degasser (MICROTRAC) under sample-specific conditions described in the following sections. Specific surface areas were calculated based on the Brunauer–Emmett–Teller (BET) model from the adsorption branches of the N_2 sorption isotherms using the instrument software. Pore size distributions (PSDs) were derived using a non-local density functional theory (NLDFT) model for cylindrical carbon pores, implemented in the instrument software.

S1.2. In situ polymerization for film preparation

Immediately prior to drop casting, silicon or glass substrates (approximately 15 mm × 15 mm) were cleaned by sequential sonication in detergent, deionized water, acetone, and isopropanol for 10 min each, followed by UV–O_3 treatment for 20 min. The cleaned substrates were transferred to an N_2-filled glove bag and immersed in a 10 mM solution of 3-aminopropyltriethoxysilane (APTES) in anhydrous toluene for 1 h. The substrates were then rinsed by sequential sonication in toluene, acetone, and isopropanol for 10 min each, and dried under a stream of N_2. Cover glass plates (approximately 20 mm × 20 mm) were cleaned by sequential sonication in acetone and isopropanol for 10 min each, followed by UV–O_3 treatment for 20 min, and then immersed in neat hexamethyldisilazane (HMDS) overnight in air. After rinsing by sequential sonication in toluene, acetone, and isopropanol, the cover plates were dried under a stream of N_2.

The precursor solutions for in situ polymerization were prepared as follows. The monomers were dissolved in a stoichiometric ratio in a mixed solvent of benzonitrile and dioxane with the aid of sonication (note: dioxane was not added in the case of TAPPy–BPDA). The resulting solution was filtered through a membrane filter to remove particulate contaminants. Distilled water and trifluoroacetic acid (TFA) were then added to achieve the desired composition. The optimized compositions for different monomer combinations are summarized in Table S1.

Table S1. Optimized compositions of precursor solutions.

Monomers (mg (μmol))		PhCN (mL)	Dioxane (mL)	H_2O (μL)	TFA (mL)	Aniline (μL)
TAPPy, 3.0 (5.2)	TA, 1.4 (10)	0.70	0.30	10	0.20	–
TAPPy, 3.0 (5.2)	DFTA, 1.8 (10)	0.70	0.30	10	0.20	–
TAPPy, 3.0 (5.2)	DMTA, 2.0 (10)	0.70	0.30	40	0.20	–
TAPPy, 3.0 (5.2)	DMBPDA, 2.8 (10)	0.70	0.30	80	0.20	–
TAPPy, 3.0 (5.2)	BPDA, 2.2 (10)	1.00	–	20	0.20	2.0
TAPB, 2.4 (6.8)	BTCA, 1.1 (6.8)	0.70	0.30	20	0.20	–

In situ polymerization was carried out either in humidity-controlled air using an acrylic box or under N_2 in a bell jar (Figure S1). The relative humidity (RH) inside the acrylic box was adjustable from approximately 5% to 70% using an AHCU-2 humidity controller (KITZ MICRO FILTER), while dry N_2 was used to approximate

0% RH conditions. At the selected humidity level, a substrate was placed on a hot plate maintained at 60 °C, and a 20 μL aliquot of precursor solution was drop-cast onto the substrate and allowed to evaporate. After evaporation of most of the solvent, residual solvent was removed by heating at 120 °C for 5 min. This drop-casting process was repeated as required. In the final casting step, the deposited solution was covered with an HMDS-treated glass plate during evaporation. The cover plate was removed once the film was visually confirmed to be dry, and the film was further dried by heating at 120 °C for 5 min. The resulting films were sequentially rinsed in THF, acetone, and MeOH, and then dried under a stream of N_2.

Amorphous TAPPy–TA films were prepared by performing the drop casting under N_2 without a cover glass, while maintaining identical substrate and film treatments as those employed for the preparation of COF films.

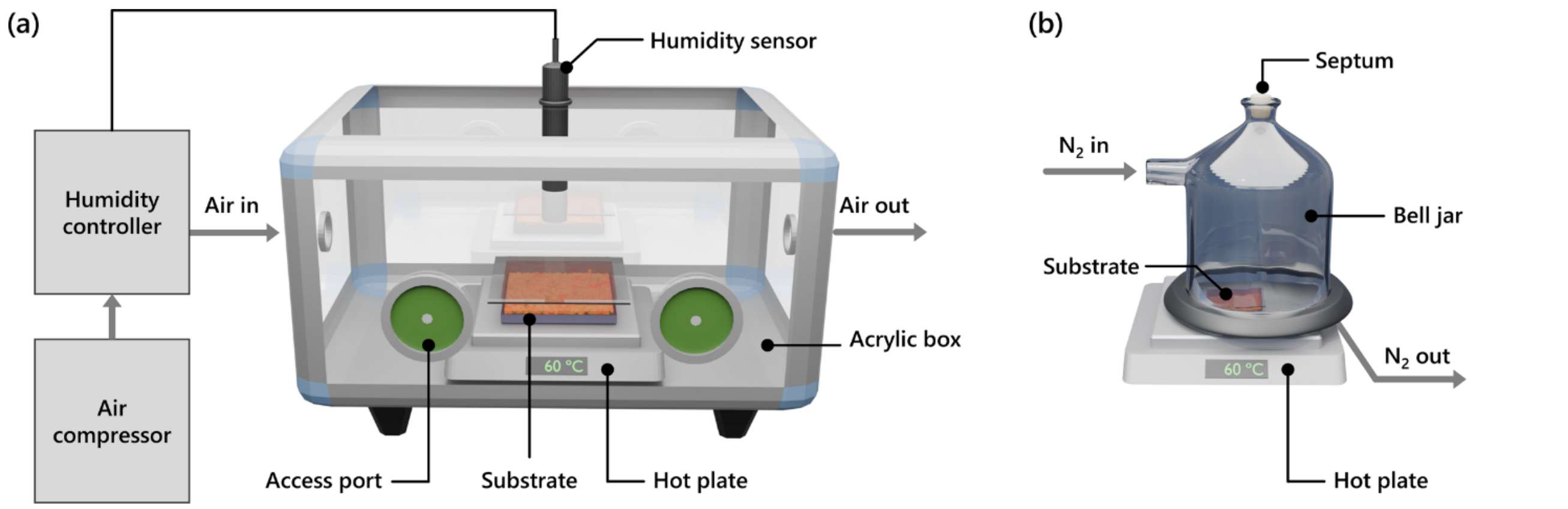

Figure S1. Schematic illustrations of the experimental setups for humidity-controlled in situ polymerization. (a) In humidity-controlled air. (b) Under N_2.

S1.3. Evaluation of moisture response of TAPPy–TA COF films

Films prepared on glass substrates were placed below a gas outlet nozzle connected to a flow line that allowed switching between dry and humidified N_2 streams (Figure S2). The gas flow rate was maintained at 2.0 L min^{-1} using mass flow controllers. The humidified N_2 was generated by bubbling dry N_2 through water in a gas-washing bottle equipped with a fritted glass diffuser.

The solvatochromic response was recorded using a smartphone camera. The video files (.mov format) were processed using a Python script, which extracted frame-by-frame color information in the RGB format using the OpenCV library and organized the data into a structured dataset with the pandas library. The color change was quantified by plotting the green-channel (G) intensity as a function of time in Figures 4a and S16b.

For chemiresistive measurements, 30-nm-thick gold electrodes were thermally evaporated onto the COF films at a rate of 0.15 Å s^{-1} under high vacuum (~10^{-3} Pa) through a shadow mask defining a channel length of 0.05 mm and a channel width of 5 mm. The electric current was monitored at an applied bias of 20 V using a B2912A source-measure unit (Agilent).

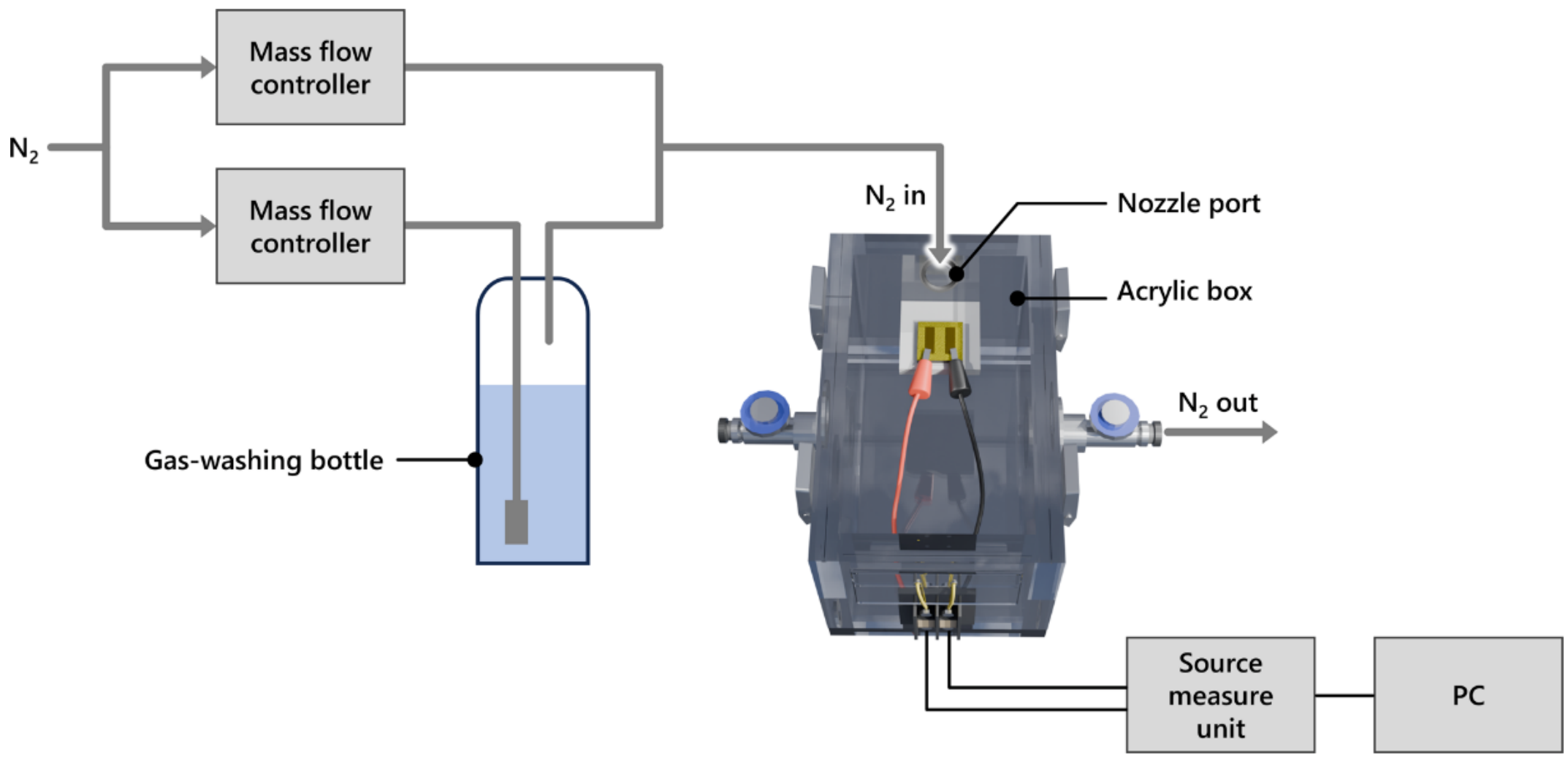

Figure S2. Schematic illustration of the experimental setup for evaluating solvatochromic and chemiresistive responses to moisture.

The "on" segments of the response profiles were fitted using a Python script employing the curve_fit function from the SciPy library and a multi-component kinetic model:

$$S(t) = S_0 + A_1\left(1 - e^{-t/\tau_1}\right) \cdot e^{-t/\tau_{\text{decay}}} + A_2\left(1 - e^{-(t/\tau_2)}\right)$$

where S_0 is the baseline current fixed to the experimentally observed value at the stimulus onset time. The first term represents a transient response characterized by rise and decay time constants (τ_1 and τ_{decay}), while the second term describes a steady-state exponential response with time constant τ_2. This bimodal kinetic model was employed to capture the non-monotonic response behavior; elucidation of the underlying mechanism will be the subject of future investigation.

The distribution of the resulting time constants was visualized using violin plots generated with Origin 2020 (OriginLab).

S1.4. Solvothermal synthesis and structural characterization of TAPPy–DMBPDA COF

The previously unreported TAPPy–DMBPDA COF was synthesized following the reported synthesis of

TAPPy–DMTA COF by Guo and coworkers, with slight modifications.[79] TAPPy (28.4 mg, 50.1 μmol), DMBPDA (27.1 mg, 100 μmol), *o*-dichlorobenzene (1.0 mL), 1-butanol (0.5 mL), and 6 M aq AcOH (0.1 mL) were added to a borosilicate glass tube (0.8 cm inner diameter, 25 cm length). The mixture was sonicated for 2 min and degassed by 3 freeze–pump–thaw cycles, and the tube was frame-sealed under vacuum (~30 Pa), resulting in an effective tube length of ~18 cm. The sealed tube was placed in a gravity oven and heated at 85 °C for 6 days to yield an insoluble solid.

The product was collected by filtration, rinsed with MeOH, and subjected to Soxhlet extraction with THF for 24 h. The solid was transferred to a screw cap vial and immersed in anhydrous EtOH. The supernatant was decanted and replaced with fresh anhydrous EtOH; this exchange was repeated three times over 24 h. The solvent was subsequently exchanged with anhydrous hexanes using the same procedure (3 cycles over 24 h). After removal of the final supernatant, the solid was dried in vacuo at 60 °C for 6 h. The isolated yield was 30.0 mg (58%, assuming complete dehydration condensation). N_2 sorption isotherms were measured immediately after vacuum drying, yielding a BET surface area of 1380 m^2 g^{-1} and a PSD peak at 2.6 nm (Figure S3a, b).

The powder X-ray diffraction (PXRD) pattern of the product was recorded on a MiniFlex600 diffractometer (Figure S3c). Pawley refinement was performed using GSAS-II over the 2θ range of 2.3–30°.[80] The background was modeled with a shifted Chebyshev polynomial function, and peak profiles were fitted using a pseudo-Voigt function. The diffraction data was well fitted in the space group *P*2/*m*, giving refined lattice parameters of a = 3.70 nm, b = 4.11 nm, c = 0.39 nm, and β = 108° with the final R_{wp} value of 3.0%.

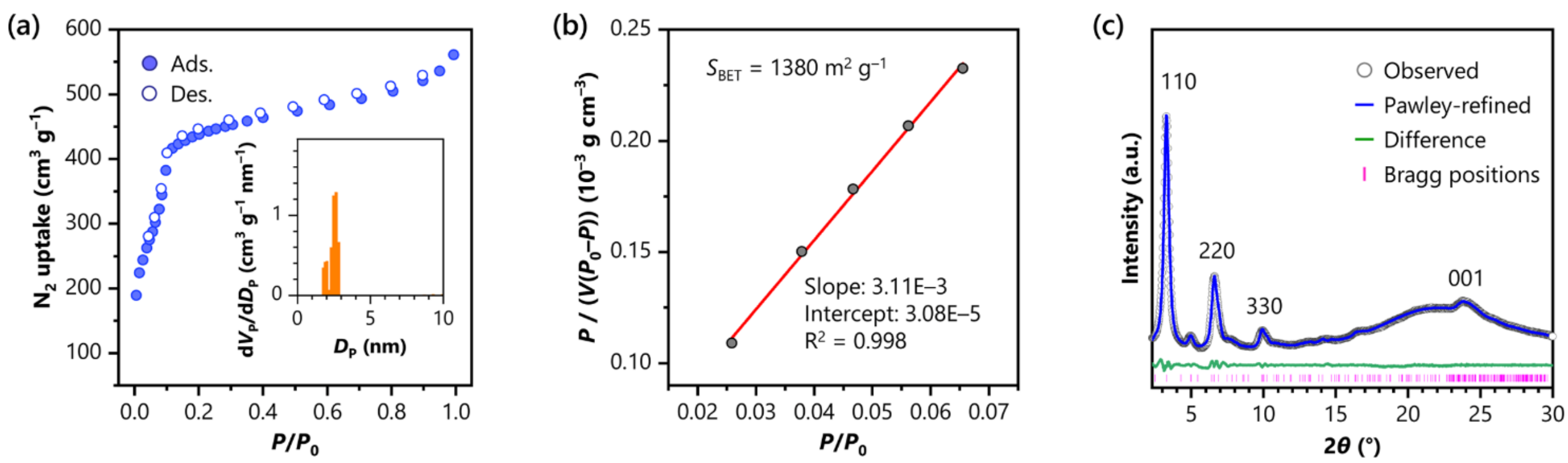

Figure S3. Structural characterization of TAPPy–DMBPDA COF. (a) N_2 sorption isotherms measured at 77 K and the corresponding PSD (inset). (b) BET plot derived from the adsorption branch of the data shown in panel a. (c) Observed and Pawley-refined PXRD patterns.

S2. Supplementary data

S2.1. Development of the film formation method

The composition of the precursor solutions was optimized to prevent premature polymer precipitation before and during film preparation (Table S1). In the case of the TAPPy–TA system as a representative example, omission of water from the optimized composition resulted in the immediate formation of dark red precipitates upon addition of TFA (Figure S4a). This observation indicates that a small amount of water effectively retards imine condensation. Similarly, replacing TFA with a weaker acid, such as acetic acid or formic acid, led to precipitation after acid addition (Figure S4b, c), suggesting that TFA functions not only as an acid catalyst but also as an aggregation suppressor.[28]

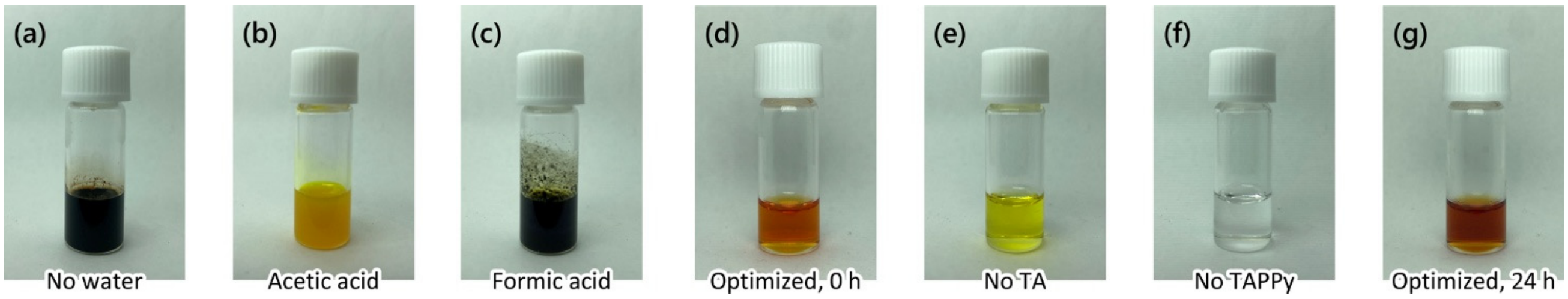

Figure S4. Optical appearances of the optimized TAPPy–TA precursor solution and its modified formulations. (a) TAPPy + TA + PhCN + dioxane + TFA. (b) TAPPy + TA + PhCN + dioxane + acetic acid + H_2O. (c) TAPPy + TA + PhCN + dioxane + formic acid + H_2O. (d) TAPPy + TA + PhCN + dioxane + TFA + H_2O, immediately after preparation. (e) TAPPy + PhCN + dioxane + TFA + H_2O. (f) TA + PhCN + dioxane + TFA + H_2O. (g) TAPPy + TA + PhCN + dioxane + TFA + H_2O, 24 h after preparation.

Note that the solution color changed from light yellow to red-orange upon addition of TFA (Figure S4d). This color change was observed only in the presence of both TA and TAPPy (Figure S4e, f), indicating partial formation of imine bonds in the precursor solution. Here, the composition of the casting solution was analyzed by ^{1}H NMR. The sample solution was prepared by dissolving the monomers TAPPy and TA in benzonitrile/1,4-dioxane-d_8/TFA-d/D_2O (70:30:20:1, v/v) at the same concentrations as those used for film preparation. While the spectrum revealed that the solution predominantly contained monomeric TAPPy and TA species, several weak signals appeared in the 8–10 ppm region after the addition of D_2O and TFA-d, consistent with partial imine formation prior to drop casting (Figure S5).

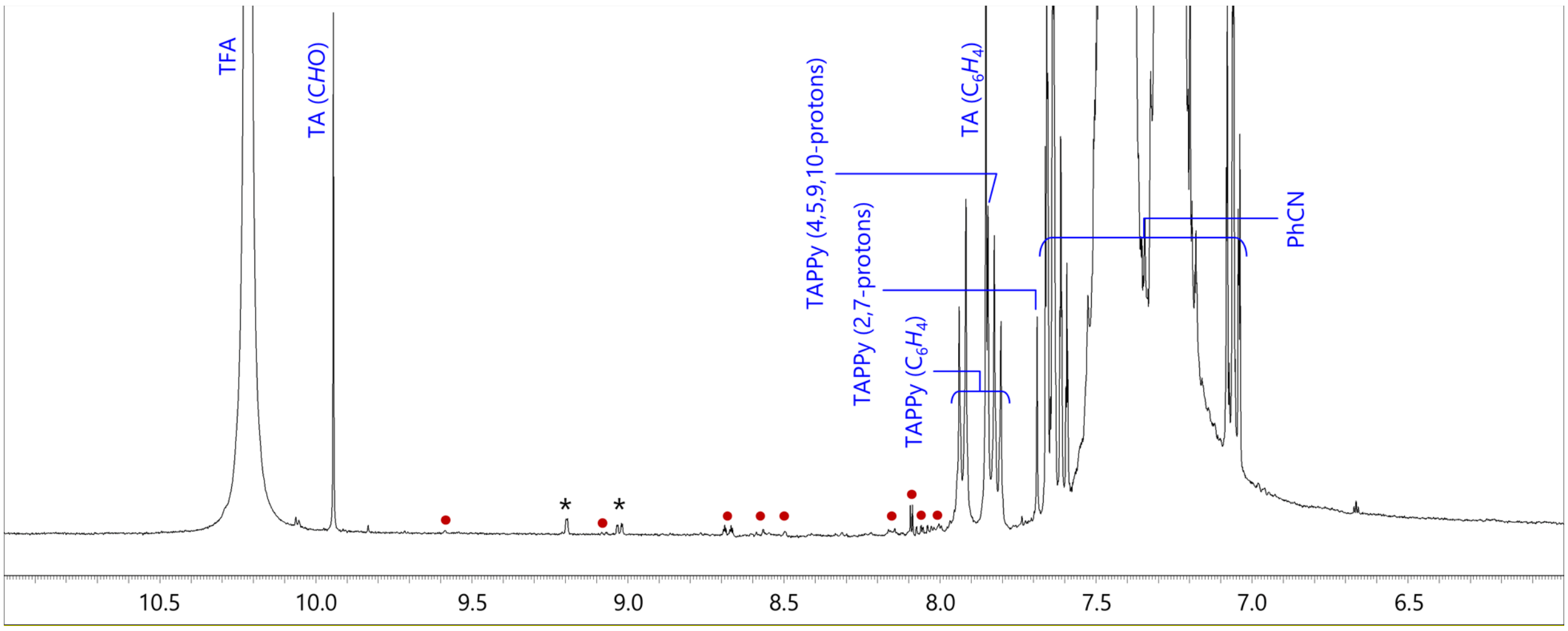

Figure S5. ^{1}H NMR of the precursor solution (400 MHz, benzonitrile/1,4-dioxane-d_8/TFA-d/D_2O (70:30:20:1, v/v)). Signals that appeared upon the addition of D_2O and TFA-d were indicated with red dots, while unknown signals that were present even before the addition of D_2O and TFA-d are marked with asterisks (*).

It is also worth noting that, although the color of the optimized solution gradually darkened over time, no precipitation was observed and the solution remained usable for at least 24 h (Figure S4g). To further monitor changes in the precursor solution during aging, UV–vis absorption spectra were recorded immediately after preparation and after 24 h. As shown in Figure S6, the aged solution exhibited a slight bathochromic shift of the absorption tail, consistent with the observed color darkening.

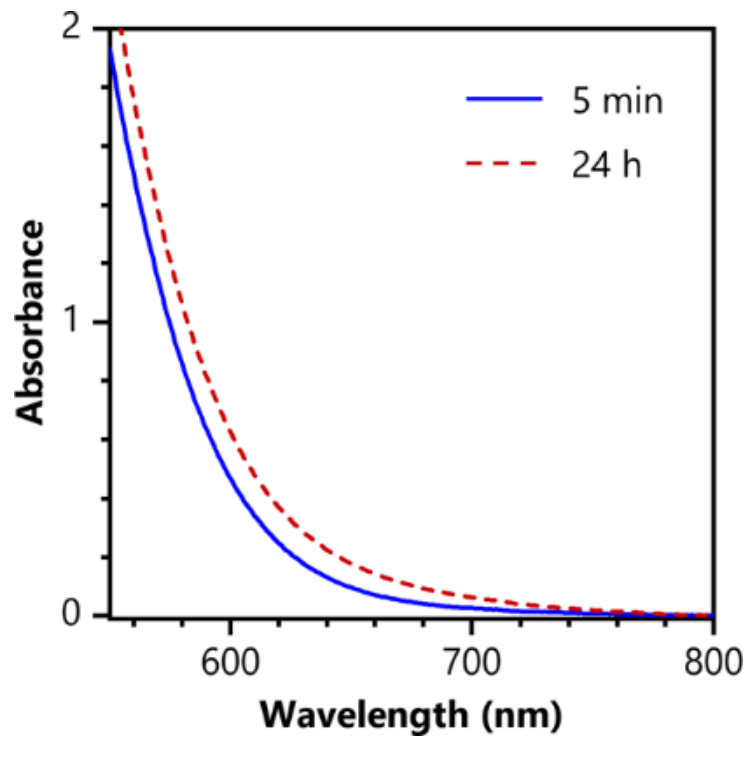

Figure S6. UV–vis absorption spectra of a TAPPy–TA precursor solution measured immediately after preparation (5min, blue solid line) and after aging (24 h, red dashed line).

The solvent evaporation temperature for the TAPPy–TA system was optimized at 60 °C. At a lower temperature (40 °C), evaporation required as long as ~4 h when a cover plate was placed over the deposited solution, compared to ~1 h at 60 °C, and resulted in significantly increased surface roughness and randomized

orientation (Figure S7). On the other hand, at a higher temperature (80 °C), no obvious difference was observed regarding film morphology as compared to the optimized temperature (Figure S7). However, the evaporation was too fast (< 2 min) and the process reproducibility decreased. The optimized evaporation temperature of 60 °C was applied to the preparation of the other COFs investigated this study.

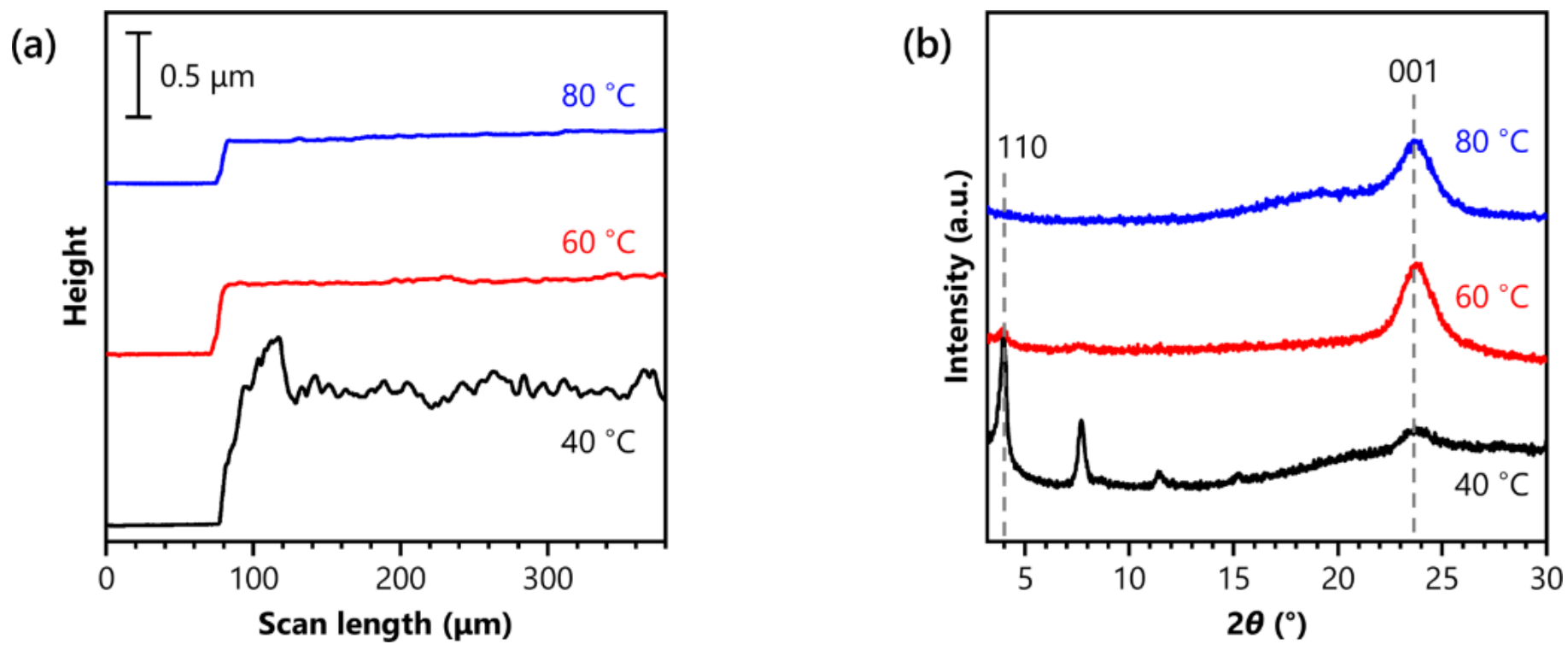

Figure S7. Structural analysis for TAPPy–TA COF films prepared at different temperatures. (a) Surface profiles measured by using a DektakXT surface profiler. (b) Out-of-plane XRD patterns measured by using a MiniFlex600 diffractometer. All films were prepared by double drop casting, and experimental conditions other than evaporation temperature were kept consistent between different samples.

The effect of a cover glass was evaluated by directly comparing TAPPy–TA films prepared with and without a cover glass. The results demonstrate that the use of a cover glass significantly improves crystallinity (Figure S8a, b). In contrast, the surface morphology remains largely unaffected (Figure S8c).

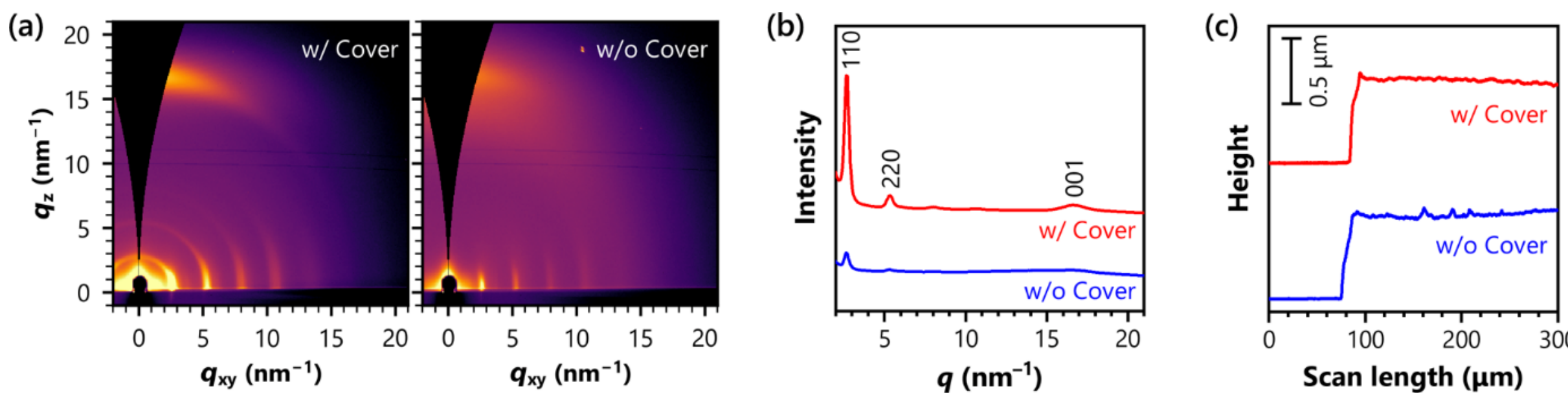

Figure S8. Effect of the cover glass on crystallinity and surface morphology. (a) 2D GIWAXS patterns of TAPPy–TA film prepared with (left) and without (right) the use of a cover glass. The films were prepared under N_2 by triple drop casting using 20 μL of precursor solution for each deposition step. (b) Azimuthally integrated intensity profiles derived from the 2D GIWAXS data in panel a. (c) Surface profiles of the corresponding films.

Regarding the number of drop-casting cycles, sharp X-ray scattering peaks were observed even with a limited number of drop-casting cycles when a cover glass was applied during the final casting step, indicating that the cover-glass treatment plays a dominant role in improving the crystallinity of the resulting COF films (Figure S9).

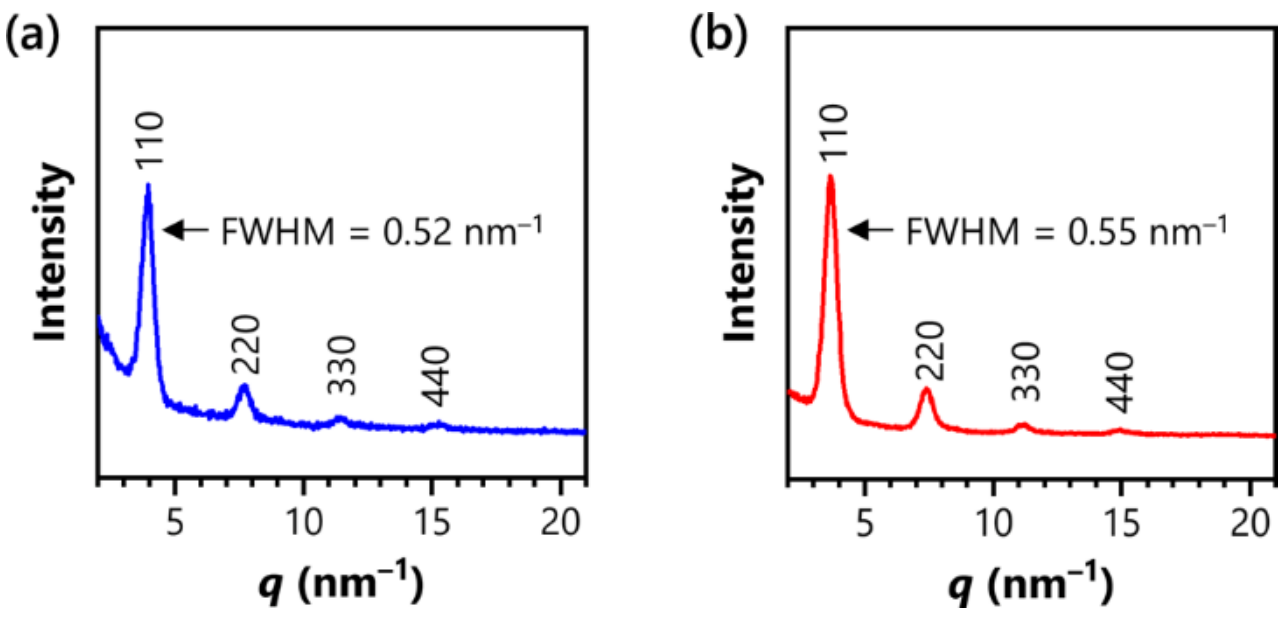

Figure S9. In-plane XRD pattern of TAPPy–TA films prepared by (a) two and (b) ten drop-casting cycles under N_2 with covering during the final solvent-evaporation step. Similar full width at half maximum (FWHM) values of the 110 peak indicate comparable structural order in the covalent network. The XRD patterns are the same as those shown in Figure 2c and Figure 3b.

S2.2. GIWAXS analysis of TAPPy–TA COF films prepared at different humidity levels

Selected TAPPy–TA COF films were characterized by 2D GIWAXS using synchrotron radiation. The film prepared under N_2 exhibited a strong scattering feature along the q_z axis at ~16 nm^{-1}, which is assigned to the 001 reflection (Figure S10a). Scattering arcs at approximately 2.6, 5.3, and 8.0 nm^{-1}, corresponding to 110, 220, and 330 reflections, respectively, were observed near the q_{xy} axis. This scattering pattern indicates that the COF crystallites preferentially adopt face-on orientation within the film.

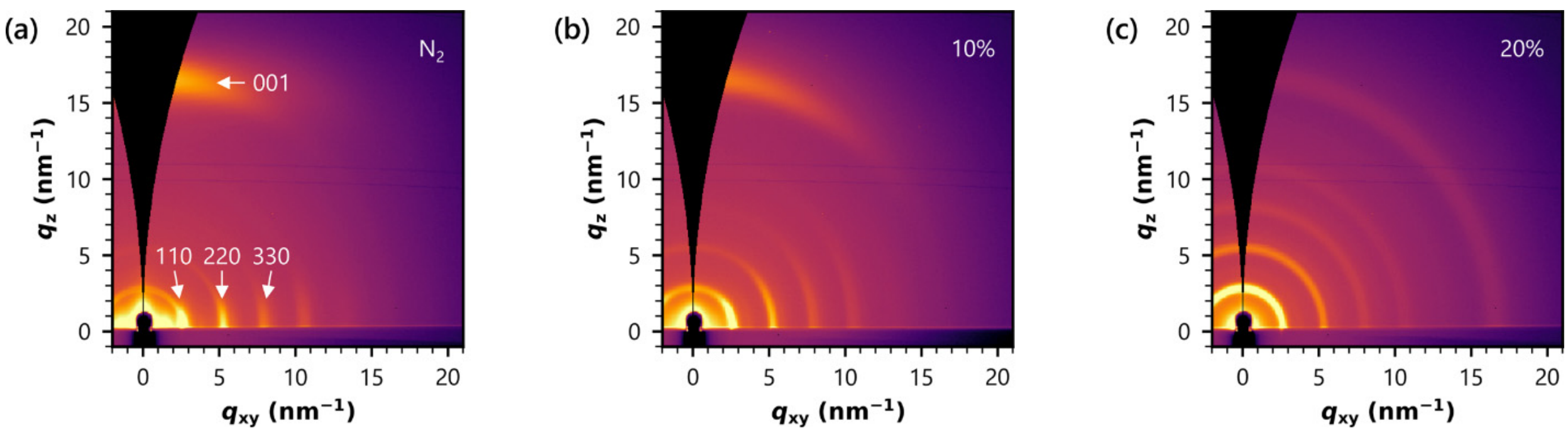

Figure S10. 2D GIWAXS patterns of TAPPy–TA COF films prepared at different humidity levels. (a) N_2. (b) 10% RH. (c) 20% RH. All films were prepared by double drop casting.

The film prepared at 10% RH showed a weaker preference for face-on orientation, whereas the film prepared at 20% RH exhibited no discernible preferred orientation, displaying nearly isotropic scattering arcs across the 2D pattern (Figure S6b, c). To quantitatively evaluate the degree of crystallite orientation, the Hermans orientation factor (OF) was calculated for the three films.[81] The calculation was performed based on the azimuthal intensity profiles of the 220 reflection ($q \approx 5$ nm^{-1}), extracted over the azimuthal angle range of $0° \leq \varphi \leq 85°$ (Figure S11). Here, the q_z axis was used as the reference for the azimuthal angle φ; consequently, an OF value of 1 represents perfect edge-on selectivity, 0 corresponds to an isotropic orientation distribution, and –0.5 indicates fully face-on selective ordering. The calculated OF values are –0.38, –0.28, and 0.05 for films prepared under N_2, at 10% RH, and at 20% RH, respectively. This trend quantitatively demonstrates that lower humidity promotes a higher degree of face-on orientation of COF crystallites, while high-humidity conditions result in isotropic crystallite orientation distribution.

Overall, the 2D GIWAXS results demonstrate a clear correlation between crystallite orientation and the humidity level during drop casting, consistent with the out-of-plane and in-plane XRD analyses described in the main text.

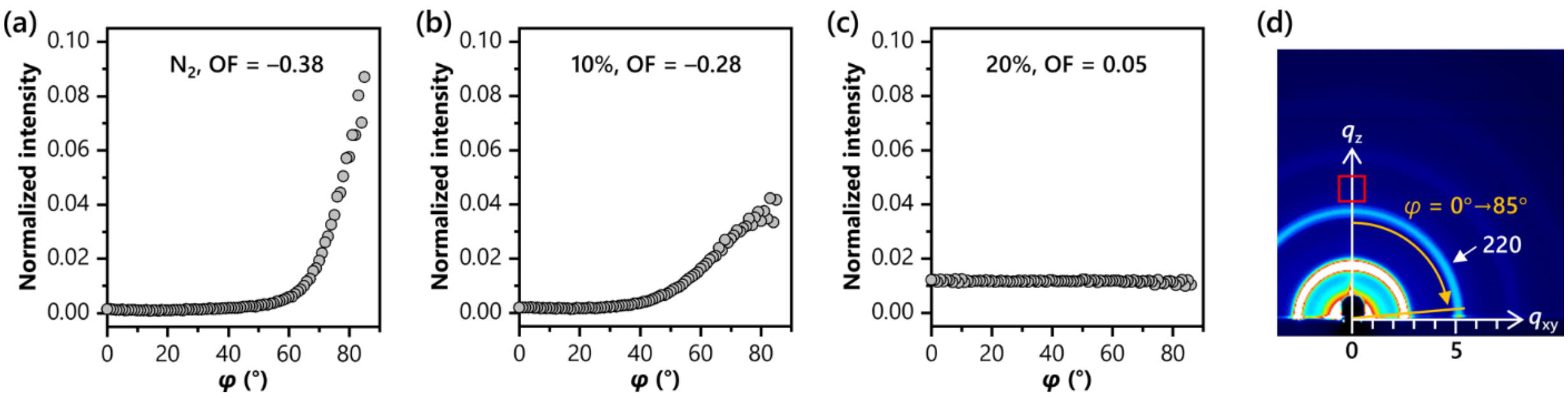

Figure S11. Crystallite orientation analysis using the 2D GIWAXS data shown in Figure S10. (a–c) Azimuthal intensity profiles of the 220 reflection for TAPPy–TA films prepared at under N_2, at 10% RH, and at 20% RH, plotted over the range of $0° \leq \varphi \leq 85°$. Intensities were normalized to the total intensity over the scanned azimuthal range. The corresponding Harmans OF values are indicated in each panel. (d) Annotated 2D GIWAXS pattern (20% RH) illustrating the azimuthal scan direction (orange arrow) and the area used for background estimation (red square). The background intensity was defined as the average scattering intensity within a 20 pixel × 20 pixel area adjacent to the 220 reflection. Hermans OF values were calculated according to the literature.[81] The high-φ region near the q_{xy} axis ($85° < \varphi \leq 90°$) was excluded from the analysis to avoid interference from the Yoneda peak and possible minor misalignment of the substrate from the ideal horizontal leveling. The intensity profiles were extracted from the raw detector images without geometric correction for the missing wedge. Accordingly, the intensity distribution near the q_z axis ($\varphi \approx 0°$) may be less accurate. Nonetheless, because the missing wedge has only a minor effect on low-q reflections, the resulting error associated with the 220 reflection ($q \approx 5$ nm^{-1}) is expected to be minimal.

S2.3. Effect of water content in the precursor solution

To unambiguously delineate the role of ambient humidity during drop casting—distinct from the contribution of the water in the precursor solution—we systematically varied the water content in the precursor solution. Precursor solutions containing 1.5- and 2.0-fold higher water content than the optimized composition were used for film fabrication under N_2, affording TAPPy–TA COF films that maintained smooth surfaces and a face-on orientation as evidenced by surface profilometry and out-of-plane XRD analysis (Figure S12). The persistence of surface morphology and preferential crystallite orientation despite substantial increases in the water content in the precursor solution indicates that ambient humidity, rather than the intrinsic water fraction in the precursor solution, is the dominant factor governing film structure.

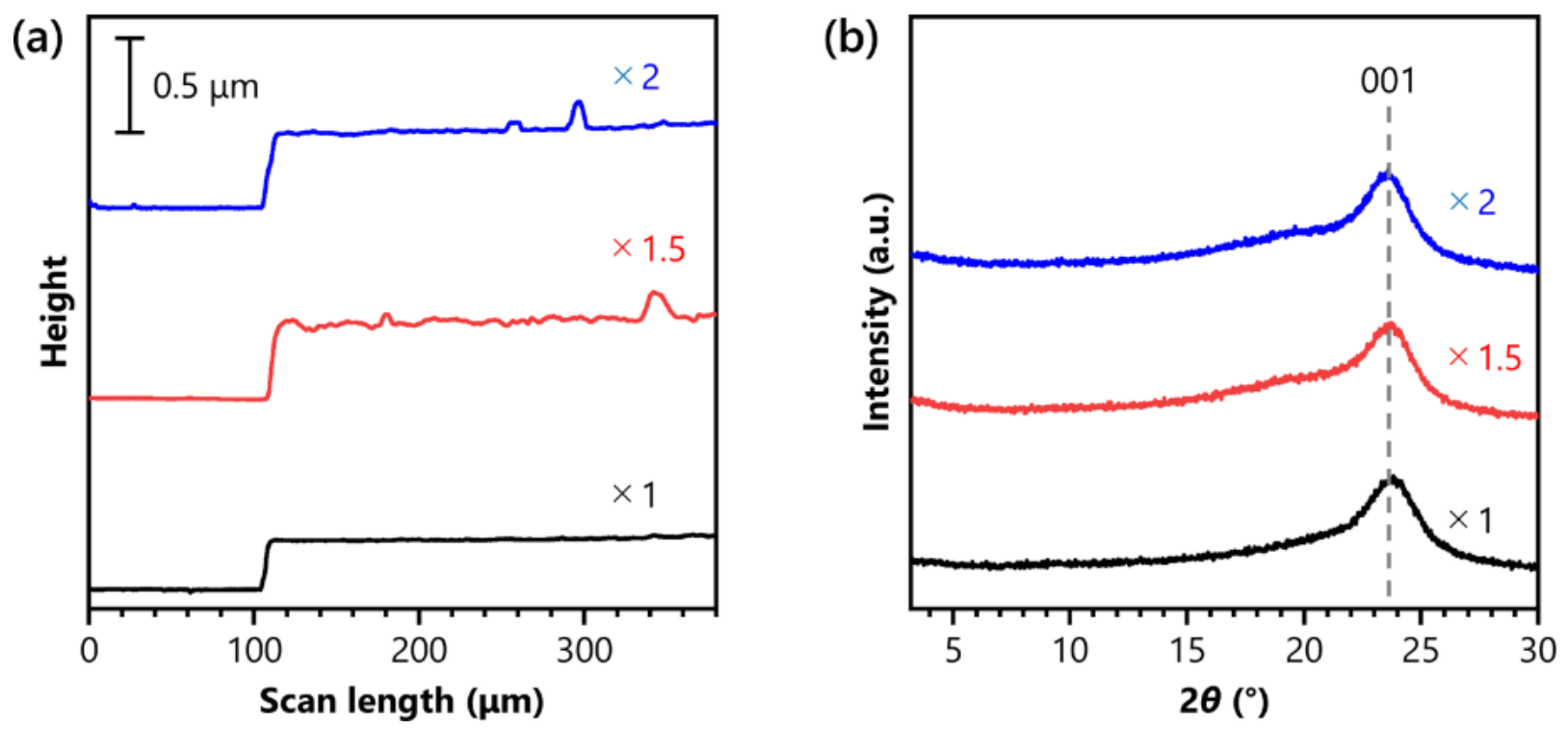

Figure S12. Structural characterization of TAPPy–TA COF films prepared from precursor solutions with different water contents (1.0-, 1.5-, and 2.0-fold of the optimized amount). (a) Surface topology profiles. (b) Out-of-plane XRD patterns measured using a MiniFlex600 diffractometer. All films were prepared by double drop casting under N_2.

S2.4. SEM and AFM characterization of TAPPy–TA COF films prepared at different humidity levels

The morphological features of TAPPy–TA COF films grown on silicon substrates under different humidity conditions were examined by SEM and AFM. Cross-sectional and surface SEM images show a clear trend of increasing surface roughness with increasing humidity level (Figure S13a, b). AFM analysis further reveals that higher humidity not only increases surface roughness but also promotes the formation of larger grains (Figure S13c). The emergence of larger grains likely leads to looser packing and less homogeneous grain boundaries, which may contribute to the broader distribution of the time constant τ_2 observed in the chemiresistive response to moisture for films prepared at 10% RH compared with those prepared under N_2.

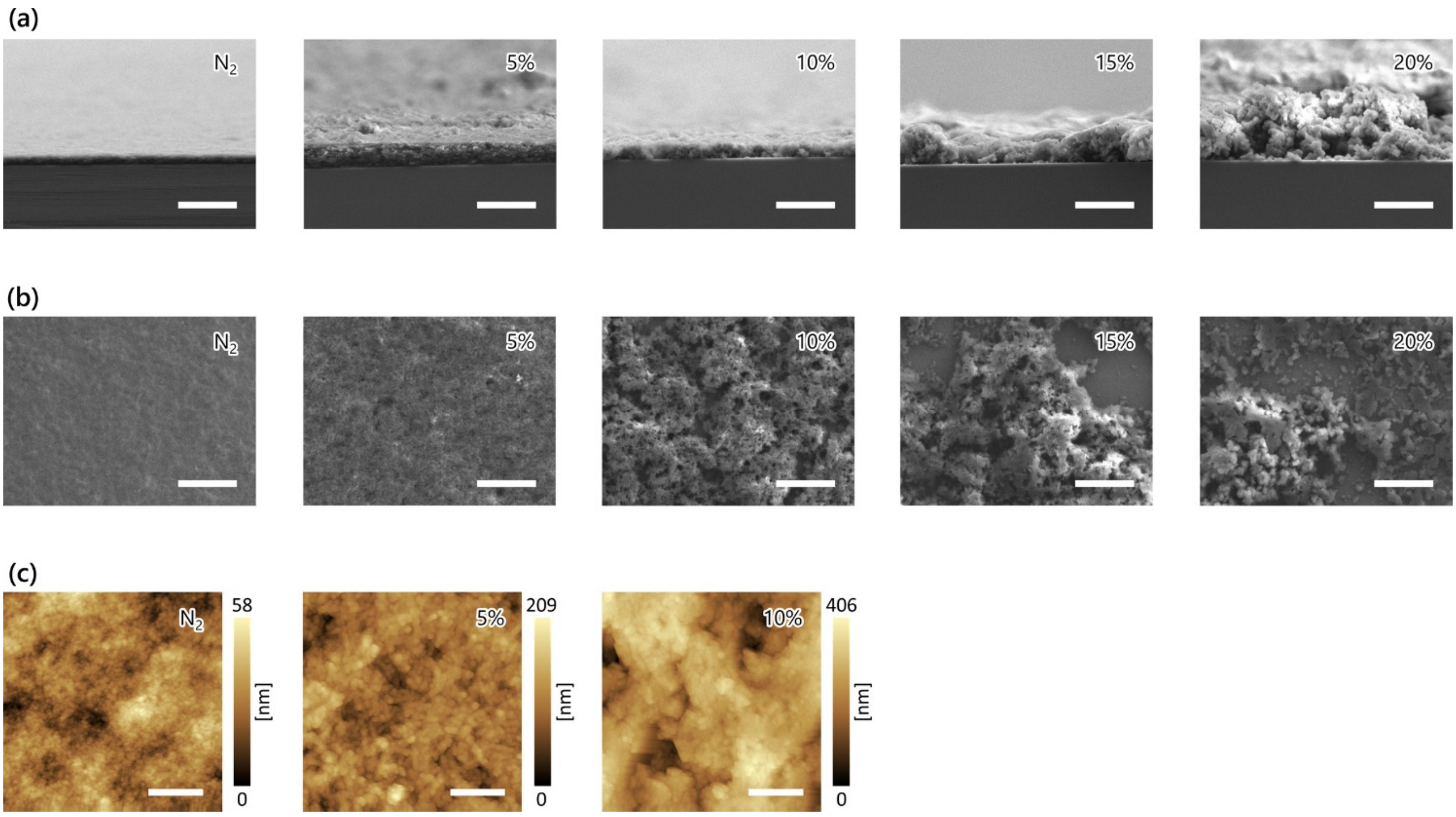

Figure S13. Microscopy analyses of TAPPy–TA COF films prepared at different humidity levels by double drop casting. (a) Cross-sectional SEM images (scale bar: 2 μm). (b) Surface SEM images (scale bar: 2 μm). (c) AFM images (scale bar: 0.5 μm). The root-mean-square roughness (R_q) values calculated for the AFM images over the entire scan area (2 μm × 2 μm) are 7.4 nm (N_2), 23.2 nm (5% RH), and 64.6 nm (10% RH). Surface roughness of the films prepared at 15% and 20% RH exceeded the vertical scan range of the microscope (1 μm); therefore, AFM images of these samples were not obtained.

S2.5. Structural characterization of humidity-guided architectures of TAPPy–TA COF films

SEM and AFM analyses showed that varying the number of drop-casting cycles (2 vs 10 cycles; ~0.3 vs ~1.6 μm thickness) did not significantly affect the surface morphology of the films. The surface SEM image of the thicker film exhibits a homogeneous and smooth surface, while the AFM image shows a uniform distribution of small grains with an R_q value of 3.6 nm (Figure S14). Combined with the cross-sectional SEM and XRD data in the main text (Figure 3a, b), these observations indicate that increasing the number of drop-casting cycles primarily increases the film thickness without inducing notable morphological changes, thereby enabling systematic thickness control.

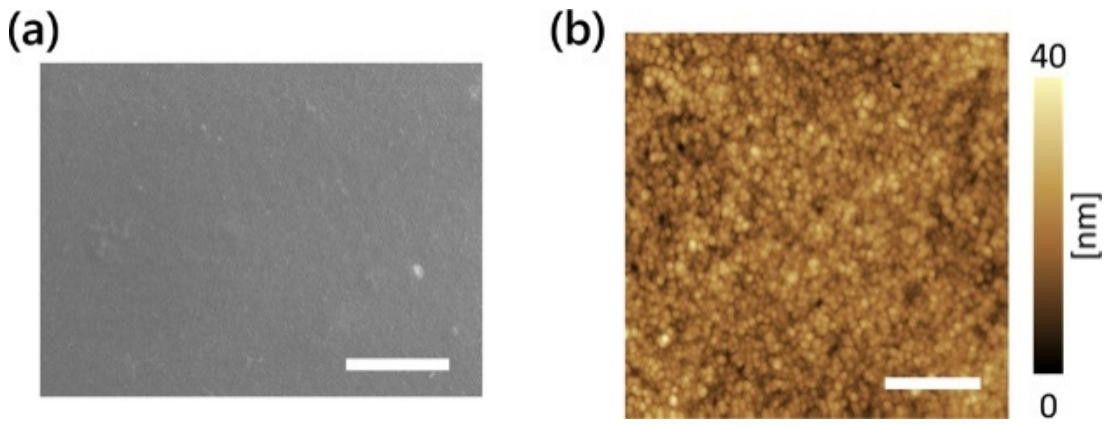

Figure S14. Microscopy analyses of a thick TAPPy–TA COF film prepared by 10 drop-casting cycles. (a) Surface SEM image (scale bar: 2 µm). (b) AFM image (scale bar: 0.5 µm). The R_q value calculated for the AFM image over the entire scan area (2 µm × 2 µm) is 3.6 nm.

Out-of-plane XRD patterns of the smooth-on-rough and rough-on-smooth TAPPy–TA COF films can both be interpreted as superpositions of face-on-oriented and non-oriented diffraction features (Figure S15). For the smooth-on-rough structure, the first morphological stratum prepared at 20% RH (rough) shows no preferred orientation. Formation of the second stratum under N_2 (smooth) leads to the appearance of a clear 001 reflection, indicating the development of face-on-oriented COF domains (Figure S15a). In contrast, for the rough-on-smooth structure, the first stratum exhibits only the 001 reflection, whereas the 110 and its higher-order reflections appear after formation of the second stratum at 20% RH (Figure S15b). Notably, the 001 reflection is more pronounced in the rough-on-smooth structure, suggesting that stacking of the 2D COF sheets becomes more organized during formation of the upper stratum.

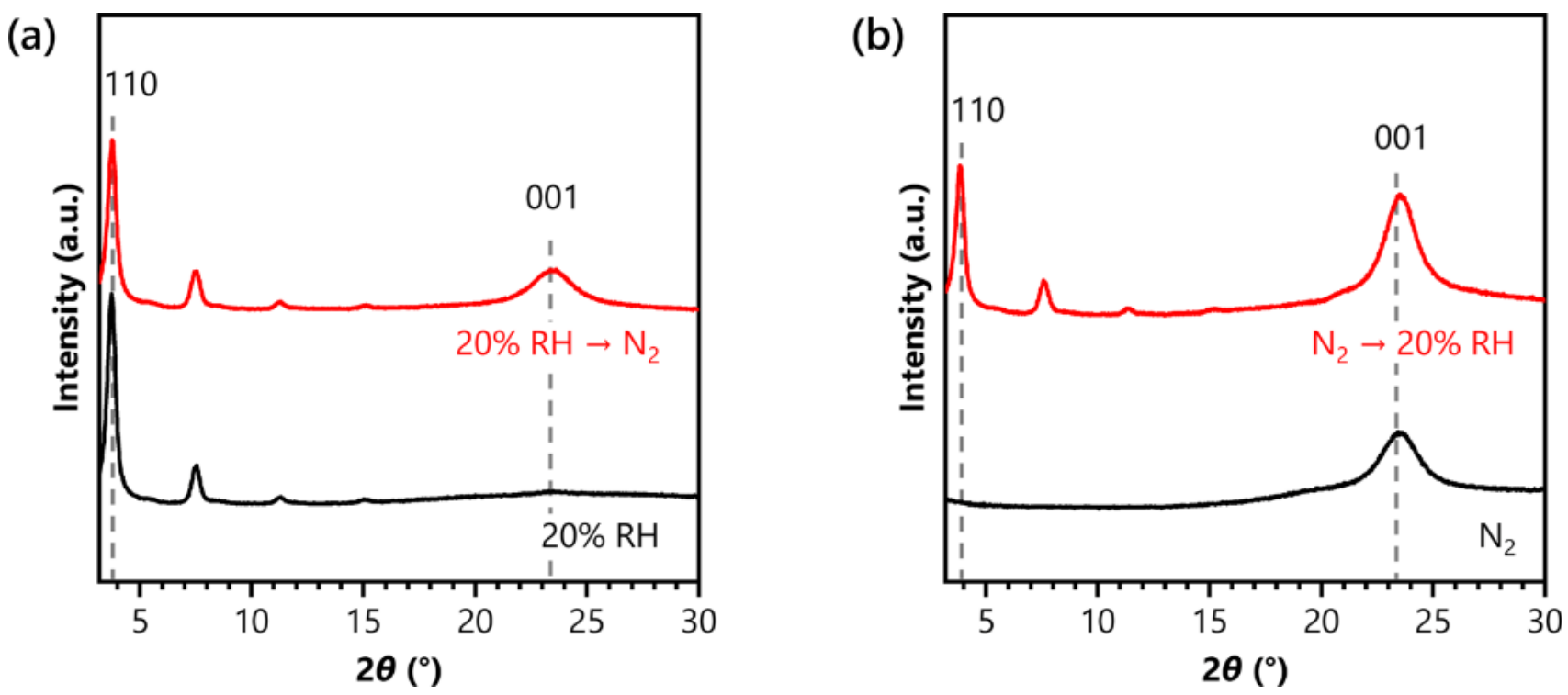

Figure S15. Out-of-plane XRD patterns of morphologically stratified TAPPy–TA COF films. (a) Smooth-on-rough structure. (b) Rough-on-smooth structure. The films were prepared by 5 drop-casting cycles at 20% RH followed by 5 cycles under N_2, or in the reverse order. The measurements were performed using a MiniFlex600 diffractometer.

S2.6. Solvatochromic response of amorphous TAPPy–TA films to moisture

Amorphous TAPPy–TA films were prepared by single drop-casting under N_2 from a precursor solution with the optimized composition (Table S1). The resulting film showed no distinct diffraction peaks in XRD, aside

from a broad hump around $2\theta = 18–25°$ in the out-of-plane measurement (Figure S16a), which is characteristic of an amorphous structure and contrasts the more defined 001 peak of the corresponding COF at about 2θ = 23.5°. This result confirms the non-crystalline nature of the film and supports the proposed film formation mechanism in which low-crystallinity polymers are formed under low RH conditions. Evaluation of this film for a solvatochromic response to moisture revealed only a minimal change in apparent color (Figure S16b). This observation indicates that the pronounced solvatochromic response observed in TAPPy–TA COF films requires an ordered framework with accessible pores that facilitate guest-molecule diffusion and incorporation. The solvatochromic inactivity of the amorphous sample of the same system was also reported by Ascherl et al.[1]

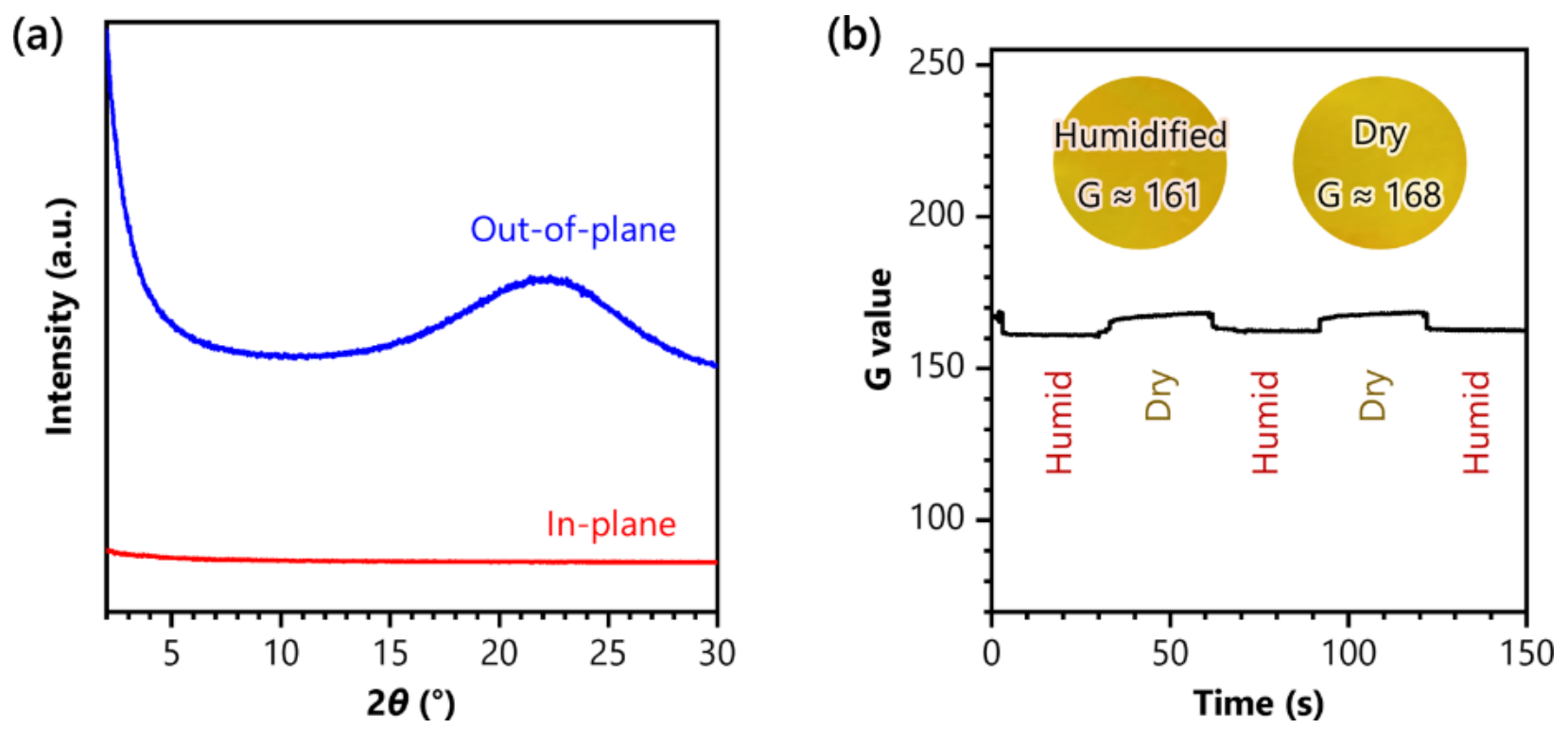

Figure S16. Characterization of amorphous TAPPy–TA films. (a) Out-of-plane and in-plane XRD patterns measured using a SmartLab diffractometer. The film was prepared on a silicon plate. (b) Change in apparent color upon exposure to moisture. The film was prepared on a glass plate.

S2.7. XRD analysis of TAPPy–TA COF powder collected from face-on oriented films

XRD analysis was conducted on a powder sample of TAPPy–TA COF obtained by scraping five face-on-oriented films from silicon substrates with the aid of MeOH to minimize sample loss from static charging. The films were prepared by 10 drop-casting cycles under N_2, with a glass cover placed over the deposited solution in the final cycle. The resulting XRD pattern exhibits distinct diffraction peaks at 2θ positions consistent with those of the film samples (Figure S17). Notably, the relative peak intensities differ from those observed in the film XRD data. One possible origin of this deviation is solvent-induced interlayer shifting during scraping with MeOH.[82] Nonetheless, the data clearly demonstrates that the crystalline covalent framework of TAPPy–TA COF is preserved after mechanical scraping from the substrate, consistent with the high porosity observed for the same material (Figure 4b in the main text). It should be also noted that the film samples were exposed to

MeOH during washing, yet their XRD peak intensity ratios remain similar to those of solvothermally synthesized samples without interlayer shifting. This difference between the film and scraped powder samples may arise from the drying procedures (film: under a stream of N_2, scraped powder: under vacuum).

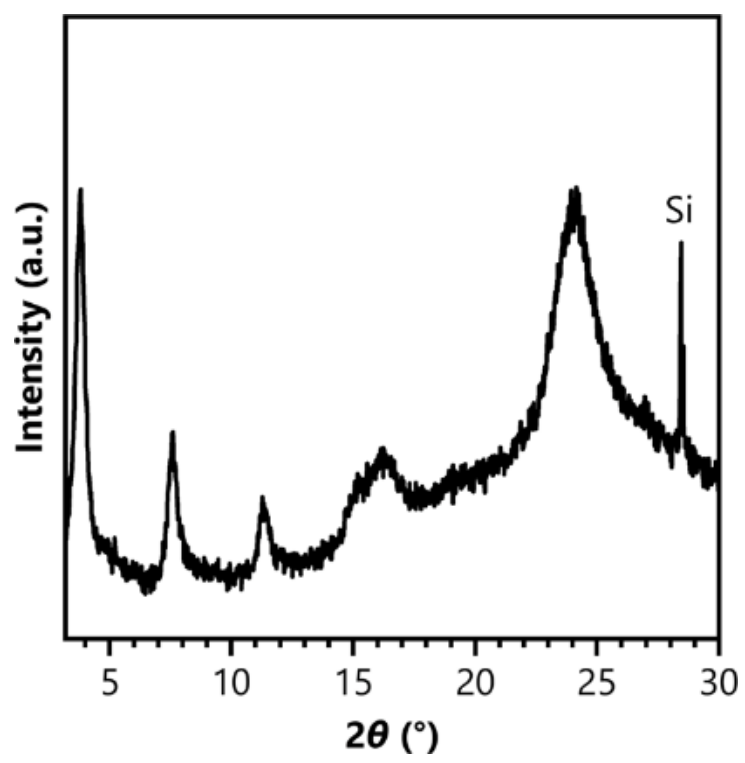

Figure S17. XRD pattern of a powder sample of TAPPy–TA COF collected by scraping face-on-oriented films from silicon substrates with the aid of MeOH. A sharp peak at $2\theta = 28.4°$ most likely originates from silicon particles introduced during scraping. The XRD data was collected using a MiniFlex600 diffractometer.

S2.8. Chemiresistive response of TAPPy–TA COF films to moisture

Representative chemiresistive response of a non-oriented TAPPy–TA COF film (prepared at 10% RH on glass by triple drop casting) upon exposure to moisture and the corresponding data analysis are shown in Figure S18. The time constants (τ) and coefficients of determination (R^2) obtained from curve fitting are summarized in Table S2, including those of the face-on-oriented films. The violin plots in Figure 5d in the main text have been generated from these values.

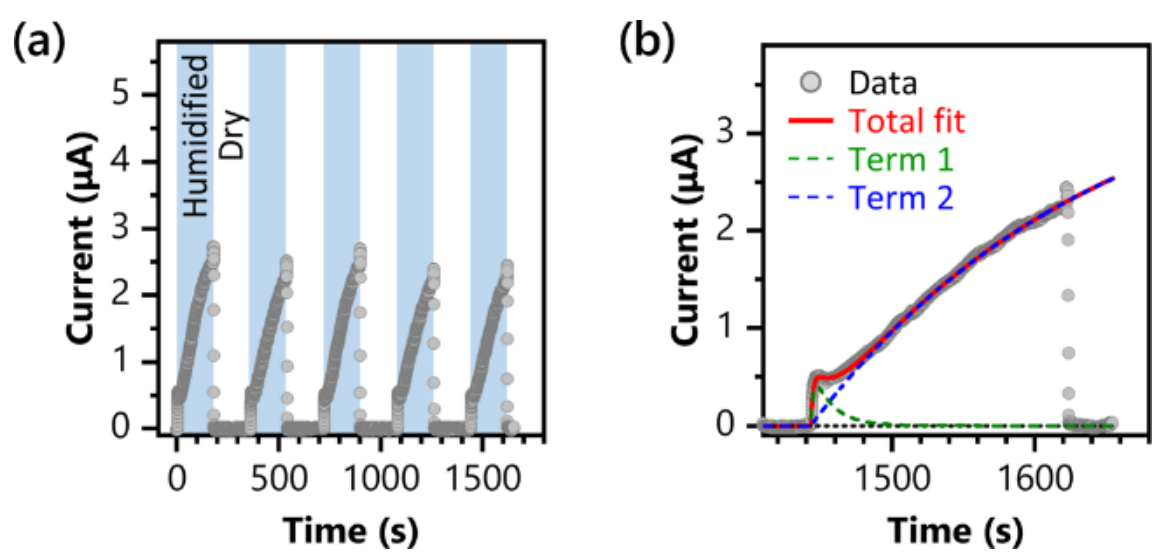

Figure S18. Chemiresistive response of a non-oriented TAPPy–TA COF film to moisture. The film was prepared at 10% RH on glass. (a) Change in electric current at an applied vias of 20 V under alternating exposure to humidified and dry N_2. (b) Representative curve fitting of an "on" segment in panel a. Fitting results: $\tau_1 = 2$ s, $\tau_{decay} = 14$ s, $\tau_2 = 199$ s, $R^2 = 0.9990$.

Table S2. Time constants (τ) and coefficients of determination (R^2) obtained from the curve fitting of the chemiresistive response data.

Face-on-oriented films prepared under N_2				
Entry	τ_1	τ_{decay}	τ_2	R^2
1	3.1	19.4	58.1	0.9996
2	4.3	15.5	71.9	0.9996
3	3.7	13.8	68.9	0.9996
4	3.1	14.3	67.5	0.9996
5	2.7	14.3	66.8	0.9996
6	3.3	37.7	124.7	0.9970
7	3.7	28.8	122.8	0.9997
8	3.3	28.0	103.8	0.9994
9	3.2	29.1	104.0	0.9995
10	3.1	29.7	96.1	0.9995
11	3.3	33.5	98.0	0.9994
12	3.5	14.7	79.2	0.9977
13	3.7	14.2	77.1	0.9992
14	3.9	15.6	81.2	0.9991
15	3.6	15.7	76.2	0.9991
16	3.7	17.1	80.9	0.9990
17	3.3	16.4	76.6	0.9993
18	2.2	9.1	153.6	0.9999
19	1.4	9.9	127.9	0.9999
20	1.2	12.8	105.7	0.9998
21	1.3	11.6	136.3	0.9998
22	1.1	10.9	108.5	0.9999
23	1.2	8.2	112.1	0.9998
24	1.2	11.7	110.9	0.9997
25	3.1	9.2	101.8	0.9986
26	1.1	25.7	59.8	0.9995
27	1.6	19.7	69.5	0.9992
28	1.6	17.1	80.4	0.9996
29	1.5	27.3	75.2	0.9981
30	2.6	17.9	73.8	0.9977
31	1.9	18.9	88.4	0.9984
32	1.0	21.0	151.1	0.9986
33	0.6	15.4	178.1	0.9996
34	0.8	17.7	109.9	0.9994
35	0.8	17.6	109.6	0.9987

Non-oriented films prepared at 10% RH				
Entry	τ_1	τ_{decay}	τ_2	R^2
1	2.2	8.1	149.5	0.9982
2	2.1	13.1	182.8	0.9988
3	3.4	8.0	177.5	0.9986
4	2.0	12.6	157.8	0.9988
5	2.1	14.1	198.6	0.9990
6	1.7	16.3	358.4	0.9991
7	1.0	13.3	171.8	0.9996
8	0.9	17.5	359.1	0.9994
9	0.8	14.9	353.1	0.9995
10	0.9	24.2	266.6	0.9990
11	0.5	26.6	445.5	0.9994

S2.9. GIWAXS analysis of a face-on oriented TAPPy–TA COF film under controlled humidity

A face-on-oriented TAPPy–TA film prepared under N_2 was analyzed by synchrotron-based 2D GIWAXS under controlled humidity to probe structural changes associated with water uptake. Comparison of the scattering patterns recorded at 10%, 30%, 50%, 70%, and 90% RH revealed that the 001 reflection shifted from q = 16.65 to 16.93 nm^{-1}, corresponding to a decrease in *d*-spacing from 0.378 to 0.371 nm (Figure S19). Upon reducing the RH back to 10%, the reflection returned to its initial position, indicating that this structural variation is reversible. This humidity-dependent structural response likely occurs in conjunction with the observed solvatochromic and chemiresistive responses of the TAPPy–TA COF to moisture. A detailed mechanistic correlation between the structural variation and the sensing behavior is beyond the scope of the present study and will be addressed in future work.

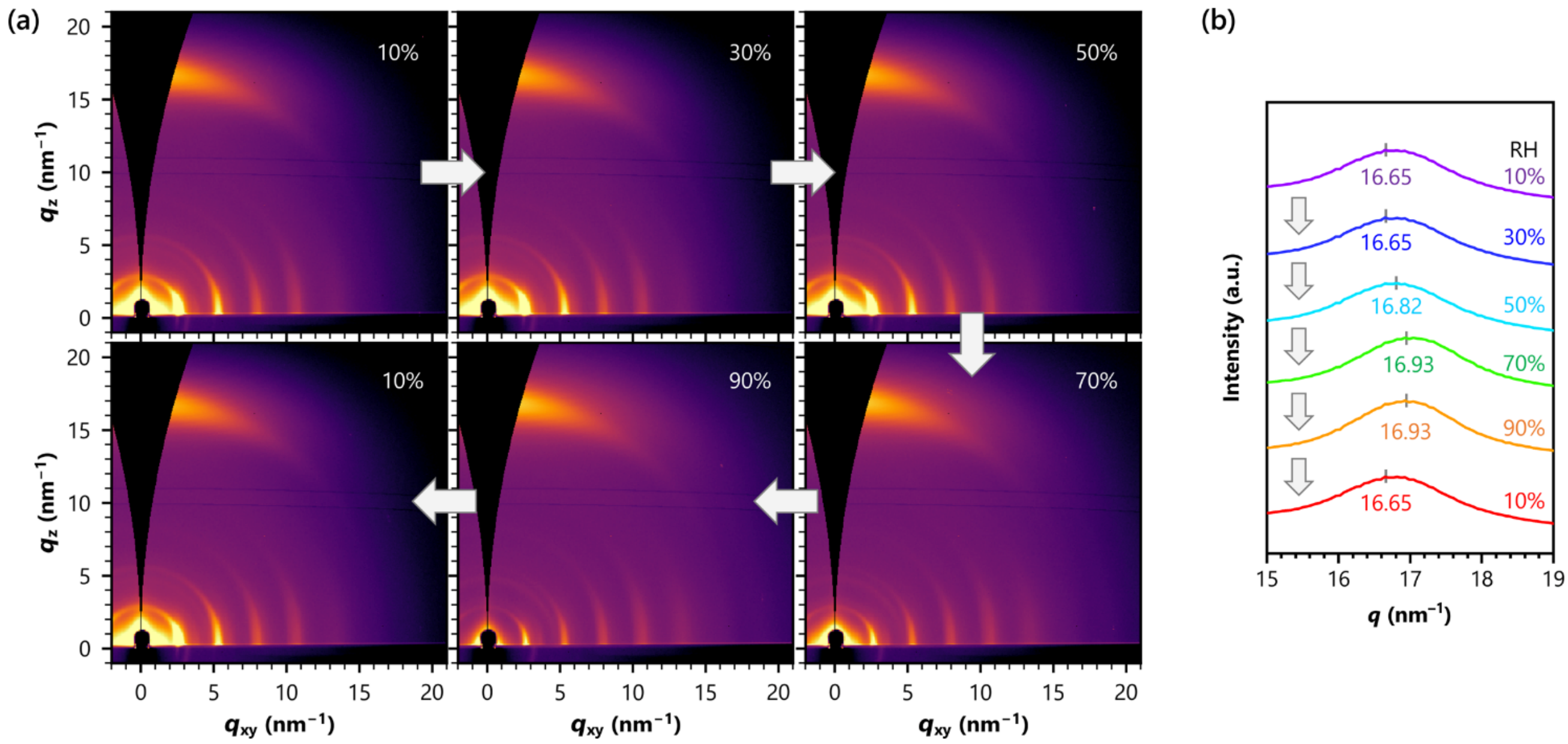

Figure S19. 2D GIWAXS analysis of a face-on oriented TAPPy–TA COF film under controlled humidity. (a) 2D scattering patterns recorded as the RH was increased from 10% to 30%, 50%, 70%, and 90%, and subsequently decreased back to 10%. (b) Azimuthally integrated intensity profiles in the q range of 15–19 nm^{-1}, highlighting the shift of the 001 reflection upon water uptake. The positions of the highest scattering intensity are indicated by tick marks with the corresponding q values. The COF film was prepared on a silicone substrate by double drop-casting under N_2.

S2.10. SEM and AFM characterization of face-on-oriented COF films other than TAPPy–TA

SEM and AFM were employed to investigate the morphology of face-on-oriented films of five 2D imine COFs: TAPPy–DMTA, TAPPy–DFTA, TAPPy–DMBPDA, TAPPy–BPDA, and TAPB–BTCA. Each film was

prepared on a silicon substrate from a precursor solution with the optimized composition listed in Table S1 under controlled humidity; RH = 40% for TAPPy–DMTA, RH = 0% (under N_2) for TAPPy–DFTA, TAPPy–BPDA, TAPB–BTCA, and RH = 70% for TAPPy–DMBPDA. Cross-sectional SEM images confirm the formation of continuous films on the substrates for all five COFs (Figure S20a). Surface SEM and AFM images of the TAPPy–DMTA film reveal irregular, needle-like features overlying an otherwise smooth layer, resulting in a moderately higher surface roughness than that of other four COFs (Figure S20b, c). Nevertheless, the overall morphology of the TAPPy–DMTA film remains substantially smoother and more homogeneous than that of TAPPy–TA films prepared at RH ≥ 10% (Figure S13). The other four COFs exhibit uniformly smooth surfaces comparable to those of the face-on-oriented TAPPy–TA film prepared under N_2. Together with the XRD results (Figure 6 in the main text), these microscopy data confirm the robustness and general applicability of the present humidity-controlled in situ polymerization for producing smooth and face-on-oriented films of 2D imine COFs.

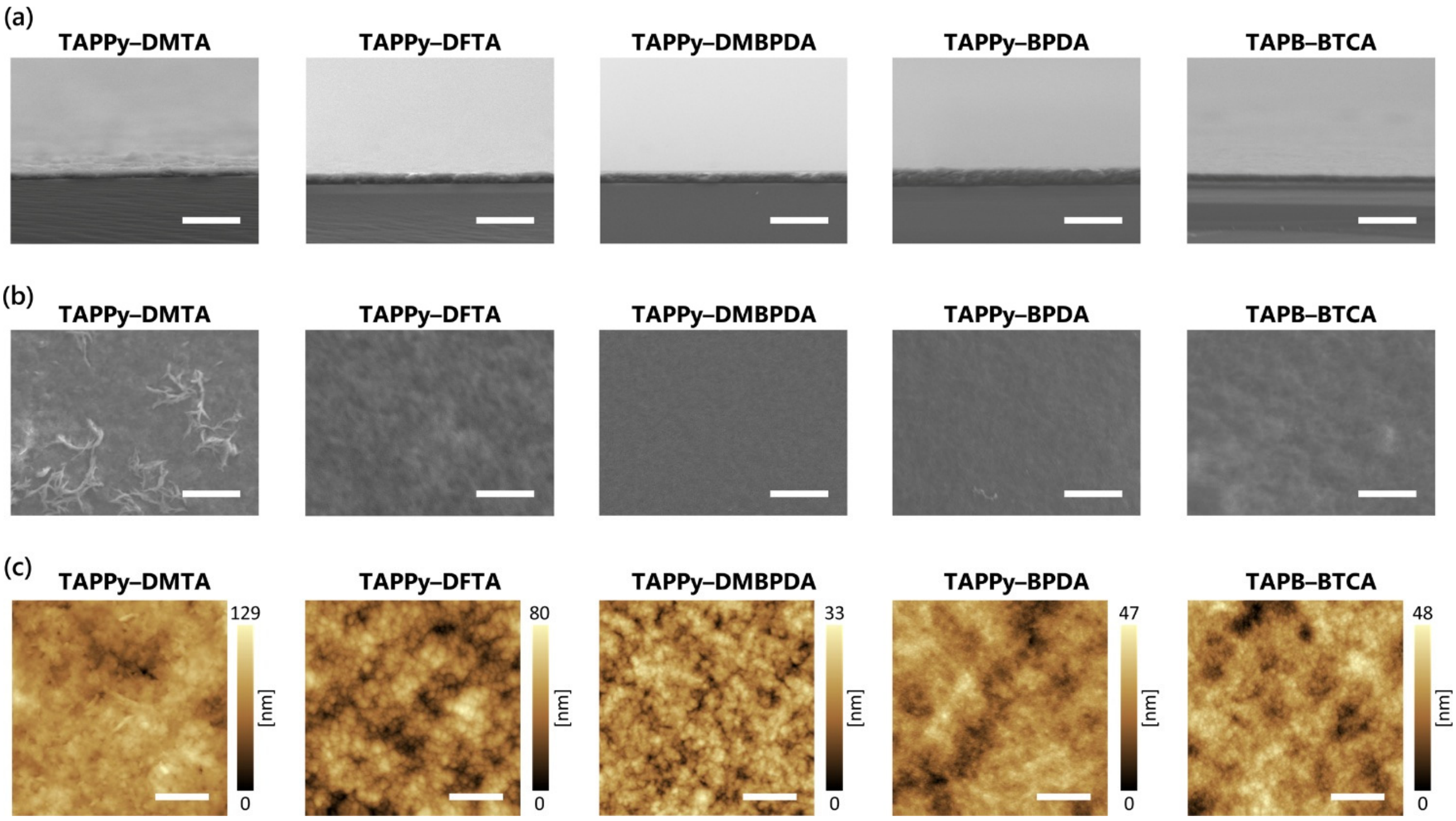

Figure S20. Microscopy characterization of face-on-oriented COF films prepared by double drop-casting on silicon substrates. The humidity during film preparation was controlled as follows: RH = 40% for TAPPy–DMTA; RH = 0% (under N_2) for TAPPy–DFTA, TAPPy–BPDA, and TAPB–BTCA; and RH = 70% for TAPPy–DMBPDA. (a) Cross-sectional SEM images (scale bar: 2 µm). (b) Surface SEM images (scale bar: 2 µm). (c) AFM images (scale bar: 0.5 µm). The R_q values calculated for the AFM images over the entire scan area (2 µm × 2 µm) are 12.9 nm (TAPPy–DMTA), 11.0 nm (TAPPy–DFTA), 4.6 nm (TAPPy–DMBPDA), 6.3 nm (TAPPy–BPDA), and 7.1 nm (TAPB–BTCA).

S2.11. Formation of low-crystallinity films with DMTA or DMBPDA

TAPPy–DMTA and TAPPy–DMBPDA COFs form highly smooth, face-on oriented films at RH levels of 40% and 70%, respectively, provided that multiple casting cycles and a cover glass are employed. To examine the possibility that low-crystallinity polymeric species are generated during the early stages of film formation under these conditions, the corresponding monomers were deposited onto silicon substrates by single drop casting at the respective RH levels. The resulting films exhibited only weak, broad humps around $2\theta = 24°$ in XRD analysis, indicating mostly amorphous or very low-crystallinity structures (Figure S21). These observations are consistent with the proposed formation of low-crystallinity intermediates and their subsequent evolution into crystalline COF structures via dynamic bond exchange.

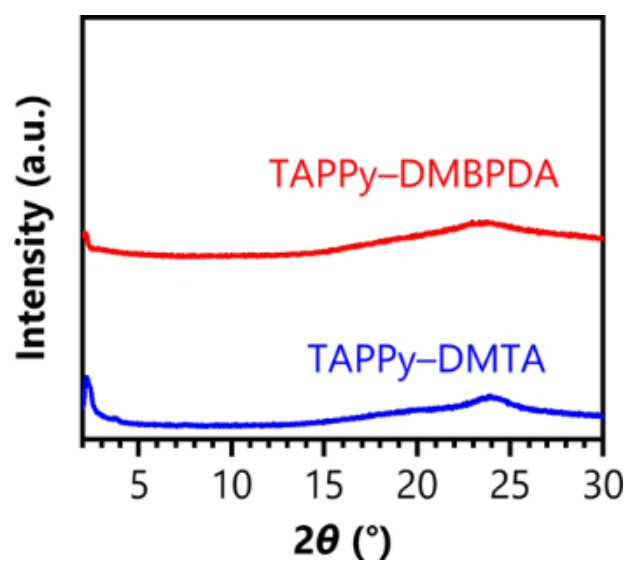

Figure S21. XRD patterns of TAPPy–DMTA and TAPPy–DMBPDA films prepared by single drop casting. The surrounding humidity during drop casting was set at 40% RH for TAPPy–DMTA and 70% RH for TAPPy–DMBPDA. The data were collected on a MiniFlex600 diffractometer.

S2.12. Porosity analysis of COF films other than TAPPy–TA

N_2 sorption analysis was performed on films of the five COFs other than TAPPy–TA, and the results are summarized in Figure S22 and Table S3. The films were prepared on glass substrates through multiple drop-casting cycles at 55% RH (TAPPy–DMBPDA) or under N_2 (other COFs) with a cover glass applied in the final evaporation step. The films were subsequently collected by scraping them from the substrates with the aid of MeOH. Prior to measurement, the samples were dried at 60 °C under vacuum for at least 6 h.

The BET surface areas obtained for the films are similar to or higher than those for bulk samples, except for TAPPy–BPDA, for which an extremely high surface area (>3000 m^2 g^{-1}) has been reported in the literature. Furthermore, the pore diameters estimated from the adsorption branches of the isotherms are close to the expected values. These results demonstrate that humidity-controlled in situ polymerization affords COF films with distinct porosity, comparable to that of their bulk counterparts in most cases.

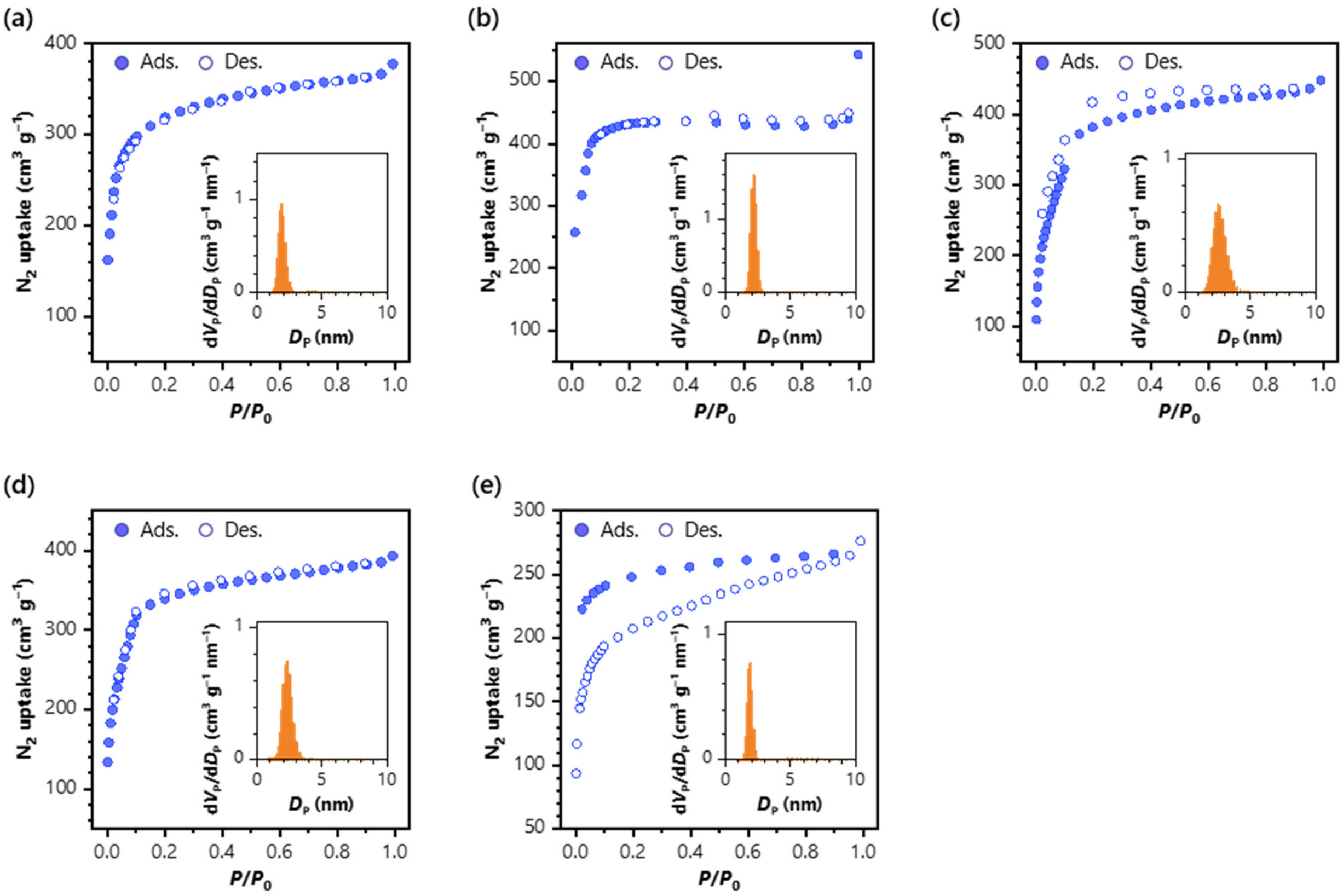

Figure S22. N_2 sorption data measured at 77 K and estimated pore size distribution (inset) of COFs deposited by the present method. (a) TAPPy–DMTA, (b) TAPPy–DFTA, (c) TAPPy–DMBPDA, (d) TAPPy–BPDA, (e) TAPB–BTCA.

Table S3. BET surfaces areas (S_{BET}) and pore size distribution peaks ($D_{p,\ peak}$) of the COF films.

COF	S_{BET} (m^2 g^{-1})		$D_{p,\ peak}$ (nm)		Reference for bulk sample data
	Film	Bulk	Film	Bulk	
TAPPy–DMTA	1200	1183	1.9	1.6	*a*
TAPPy–DFTA	1650	1435	2.2	2.9	*b*
TAPPy–DMBPDA	1500	1380	2.5	2.6	This work[c]
TAPPy–BPDA	1520	3312	2.3	2.6	*d*
TAPB–BTCA	790	1232	1.9	1.7	*e*

[a]*Nat. Commun.* **2022**, *13*, 1770. [b]*Appl. Catal. A, General* **2023**, *662*, 119269. [c]Figure S3, [d]*Chem. Sci.* **2022**, *13*, 9655. [e]*J. Am. Chem. Soc.* **2022**, *144*, 7489.

Supplementary references

[79] M. Guo, S. Jayakumar, M. Luo, X. Kong, C. Li, H. Li, J. Chen, and Q. Yang, "The Promotion Effect of π-π Interactions in Pd NPs Catalysed Selective Hydrogenation," *Nature Communications* **13** (2022): 1770, https://doi.org/10.1038/s41467-022-29299-0.

[80] B. H. Toby, and R. B. Von Dreele, "GSAS-II: The Genesis of a Modern Open-Source All Purpose Crystallography Software Package," *Journal of Applied Crystallography* **46** (2013): 544–549, https://doi.org/10.1107/S0021889813003531.

[81] J. C. Fischer, C. Li, S. Hamer, L. Heinke, R. Herges, B. S. Richards, and I. A. Howard, "GIWAXS Characterization of Metal–Organic Framework Thin Films and Heterostructures: Quantifying Structure and Orientation," *Advanced Materials Interfaces* **10** (2023): 2202259, https://doi.org/10.1002/admi.202202259.

[82] C. Kang, Z. Zhang, V. Wee, A. K. Usadi, D. C. Calabro, L. S. Baugh, S. Wang, Y. Wang, and D. Zhao, "Interlayer Shifting in Two-Dimensional Covalent Organic Frameworks," *Journal of the American Chemical Society* **142** (2020): 12995–13002, https://doi.org/10.1021/jacs.0c03691.
